# Liquid‐Phase Cyclohexene Oxidation with O_2_ over Spray‐Flame‐Synthesized La_1−*x*_Sr_*x*_CoO_3_ Perovskite Nanoparticles

**DOI:** 10.1002/chem.202103381

**Published:** 2021-10-14

**Authors:** Julia Büker, Baris Alkan, Sonia Chabbra, Nikolai Kochetov, Tobias Falk, Alexander Schnegg, Christof Schulz, Hartmut Wiggers, Martin Muhler, Baoxiang Peng

**Affiliations:** ^1^ Laboratory of Industrial Chemistry Ruhr-University Bochum Universitätsstraße 150 44780 Bochum Germany; ^2^ IVG, Institute for Combustion and Gasdynamics–Reactive Fluids and CENIDE Center for Nanointegration University of Duisburg-Essen Carl-Benz-Straße 199 47057 Duisburg Germany; ^3^ Max Planck Institute for Chemical Energy Conversion Stiftstraße 34–36 45470 Mülheim an der Ruhr Germany

**Keywords:** Cyclohexene hydroperoxide, flame-spray synthesis, in situ ATR-IR, oxygen vacancies, spin trap EPR, Sr doping

## Abstract

La_1−*x*_Sr_*x*_CoO_3_ (*x*=0, 0.1, 0.2, 0.3, 0.4) nanoparticles were prepared by spray‐flame synthesis and applied in the liquid‐phase oxidation of cyclohexene with molecular O_2_ as oxidant under mild conditions. The catalysts were systematically characterized by state‐of‐the‐art techniques. With increasing Sr content, the concentration of surface oxygen vacancy defects increases, which is beneficial for cyclohexene oxidation, but the surface concentration of less active Co^2+^ was also increased. However, Co^2+^ cations have a superior activity towards peroxide decomposition, which also plays an important role in cyclohexene oxidation. A Sr doping of 20 at. % was found to be the optimum in terms of activity and product selectivity. The catalyst also showed excellent reusability over three catalytic runs; this can be attributed to its highly stable particle size and morphology. Kinetic investigations revealed first‐order reaction kinetics for temperatures between 60 and 100 °C and an apparent activation energy of 68 kJ mol^−1^ for cyclohexene oxidation. Moreover, the reaction was not affected by the applied O_2_ pressure in the range from 10 to 20 bar. In situ attenuated total reflection infrared spectroscopy was used to monitor the conversion of cyclohexene and the formation of reaction products including the key intermediate cyclohex‐2‐ene‐1‐hydroperoxide; spin trap electron paramagnetic resonance spectroscopy provided strong evidence for a radical reaction pathway by identifying the cyclohexenyl alkoxyl radical.

## Graphical abstract

**A complex for a complex reaction**: La_1−*x*_Sr_*x*_CoO_3_ nanoparticles were prepared by spray‐flame synthesis and applied in the liquid‐phase oxidation of cyclohexene with molecular O_2_. An optimum Sr doping of 20 at. % – with balanced amounts of oxygen vacancies and Co^2+^ cations – was found to be beneficial for cyclohexene conversion. In situ ATR‐IR and EPR spectroscopy verified the complex radical reaction network.

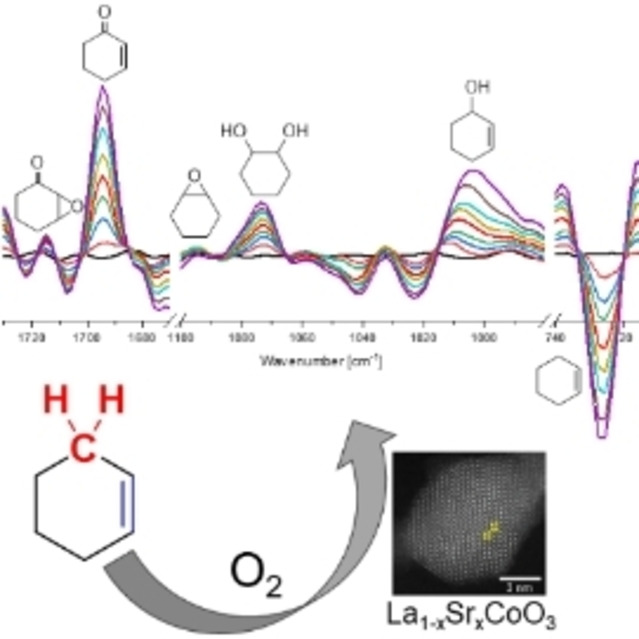

## Introduction

Heterogeneously catalyzed cyclohexene oxidation has the potential to replace industrially established synthetic routes to several important fine chemicals, such as adipic acid, by environmentally friendly processes.[^1^, ^2^] Today, adipic acid, a key intermediate in nylon‐6,6 polyamide production with a capacity of 10^6^ t per year, is produced by nitric acid oxidation of K/A oil.^[3]^ The N_2_O emission from this process measurably contributes to global warming.^[4]^ Hence, the development of alternative production routes of oxidation products that are less harmful to the environment is of high importance. Aerobic oxidation of cyclohexene offers an attractive and sustainable route by replacing the application of hazardous stoichiometric oxidants.^[5]^ Molecular O_2_ is the ultimate green oxidant, because it is inexpensive, abundant, and environmentally friendly.[^1^, ^6^]

**Scheme 1:**
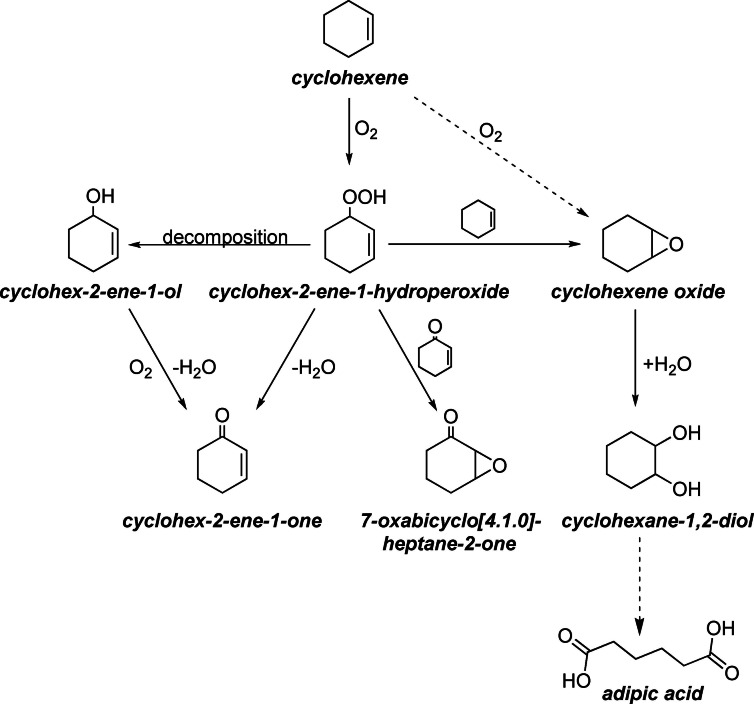
Reaction network for the oxidation of cyclohexene.^[1]^

The selective oxidation of cyclohexene is very challenging due to the presence of multiple reactive centres and its autocatalytic nature.^[7]^ The oxidation at the allylic C−H bond initially leads to the formation of the reaction intermediate cyclohex‐2‐ene‐1‐hydroperoxide, which can subsequently decompose into cyclohex‐2‐ene‐1‐ol and cyclohex‐2‐ene‐1‐one (Scheme 1). Additionally, cyclohex‐2‐ene‐1‐ol can be further oxidized to cyclohex‐2‐ene‐1‐one. Both cyclohex‐2‐ene‐1‐ol and cyclohex‐2‐ene‐1‐one are important intermediates in the manufacture of spices, medication, pesticides and insect pheromones.[^7^, ^8^] Another reaction product of the allylic pathway is 7‐oxabicyclo[4.1.0]heptan‐2‐one, formed by the oxidation of cyclohex‐2‐ene‐1‐one with cyclohex‐2‐ene‐1‐hydroperoxide.^[9]^ Direct epoxidation at the olefinic C=C double bond yields cyclohexene oxide, which is an important intermediate in coatings industry.[^7^, ^10^] Simultaneously, cyclohexene oxide can be formed by cyclohex‐2‐ene‐1‐hydroperoxide reacting with another cyclohexene molecule. Hydrolysis of cyclohexene oxide yields cyclohexane‐1,2‐diol, which represents the key intermediate of cyclohexene oxidation to form adipic acid at last. Furthermore, cyclohexane‐1,2‐diol preparation and its downstream products involve many additional application fields besides polymer chemistry such as agricultural, food, and medical chemistry.^[11]^ As the reaction network of cyclohexene oxidation using molecular O_2_ is very complex and underlies an autocatalytic reaction mechanism, reaching a high selectivity to a single major product is rather challenging.[^1c^, ^8b^] Actually, the role of the catalyst is mainly to decompose the hydroperoxide intermediate selectively.

Transition‐metal‐based perovskite oxides have attracted great attention as heterogeneous catalysts due to their flexible crystal structure, tuneable electronic configuration, and high chemical versatility.^[12]^ Perovskite‐type oxides have the general chemical formula ABO_3_, in which the A‐site is occupied by lanthanides or alkaline earth metals and the B‐site by transition metals. The large A‐site cations improve the catalyst stability, while the B‐site cations are mainly responsible for the catalytic properties, which can be further optimized by partially substituting the A‐ or B‐site cations, resulting in structural modifications related to changes of the valence state of the original cations and the formation of oxygen vacancies. Among these oxides, La_1−*x*_Sr_*x*_CoO_3_‐based perovskites were applied in a large number of heterogeneous catalytic reactions, such as electrocatalytic oxidation,^[13]^ NO_*x*_ ^[14]^ and CO oxidation.^[15]^ In these perovskites, the substitution of La^3+^ with Sr^2+^ cations is used as a method to tailor the concentration of oxygen vacancies and to generate mixed Co oxidation states.^[16]^ Using this method, Mefford et al. showed that not only surface oxygen but also mobile lattice oxygen in oxygen‐deficient La_1−*x*_Sr_*x*_CoO_3−δ_ participates in catalytic oxidation reactions like water oxidation.^[17]^ On the other hand, the introduction of Sr^2+^ into the La^3+^ site increases the basicity of the perovskite surface,^[18]^ which was stated to be advantageous for activating the oxidant.^[19]^ Thus, La_1−*x*_Sr_*x*_CoO_3−δ_ has the potential to catalyze complex oxidation reactions including cyclohexene oxidation.

The synthesis of nanoscale perovskites aims at producing catalytically active nanoparticles with high specific surface area. In this respect, the Pechini method,^[20]^ co‐precipitation,^[21]^ nanocasting techniques^[22]^ and sol‐gel methods^[23]^ were widely used. In the last two decades, spray‐flame synthesis processes were well developed for producing perovskite nanoparticles with tunable phase and chemical composition, morphology, and particle sizes over a wide range.^[24]^ Perovskite compounds synthesized by this method include LaCoO_3_, LaFeO_3_,^[25]^ LaCo_1−*x*_Fe_*x*_O_3_,^[26]^ La_0.6_Sr_0.4_CoO_3−δ_,^[27]^ La_0.6_Sr_0.4_Co_0.8_Fe_0.2_O_3−δ_ as well as Ba_0.5_Sr_0.5_Co_0.8_Fe_0.2_O_3−δ_.^[28]^ Yet, in these spray‐flame‐made Sr‐substituted perovskites, the as‐synthesized nanoparticles contain high amounts of (La,Sr)_2_CoO_4_ secondary phases and the specific surface areas are lower than 50 m^2^ g^−1^. Furthermore, these perovskite oxides were investigated only as cathode materials for the intermediate temperature solid oxide fuel cell (SOFC) rather than for heterogeneous catalysis. Thus, it is of great interest to synthesize phase‐pure, high surface area La_1−*x*_Sr_*x*_CoO_3_ nanoparticles containing different Sr amounts and to determine their catalytic properties in cyclohexene oxidation in the liquid phase.

In the present work, we report for the first time on the liquid‐phase oxidation of cyclohexene with molecular O_2_ over a series of spray‐flame‐synthesized La_1−*x*_Sr_*x*_CoO_3_ perovskite nanoparticles under mild conditions, aiming at understanding the complex reaction network and the effects of Sr substitution on the structural and catalytic properties. Therefore, bulk, morphological and surface properties of the La_1−*x*_Sr_*x*_CoO_3_ nanoparticles were characterized, and their catalytic performance was systematically investigated. Low temperature X‐band continuous wave (cw) electron paramagnetic resonance (EPR) spectroscopy was employed for the detection of paramagnetic Co states in La_1−*x*_Sr_*x*_CoO_3_ with different Sr doping levels. EPR information on the magnetic La_1−*x*_Sr_*x*_CoO_3_ properties was further complemented by temperature‐dependent superconducting quantum interference device (SQUID) magnetization measurements. The influences of initial cyclohexene concentration, pressure, and temperature were studied to obtain information about the reaction kinetics of cyclohexene oxidation over La_0.8_Sr_0.2_CoO_3_ (Sr20). Furthermore, a reusability test over Sr20 was performed. In situ attenuated total reflection infrared (ATR‐IR) spectroscopy was used to probe cyclohexene oxidation and to verify the complex reaction network. In addition, room temperature spin trap EPR was applied to detect and characterize radical species and to assign free radical reaction pathways.

## Results and Discussion

### Catalyst characterization

Using a defined La/Sr/Co precursor ratio in the precursor solution, perovskite nanoparticles containing La, Sr, and Co were produced by spray‐flame synthesis. Based on the molar fraction of Sr precursor used in the spray‐flame synthesis, that is, xSr, Sr‐containing materials were denoted “Sr(x)” and the sample containing only La and Co was labeled LCO.

**Figure 1:**
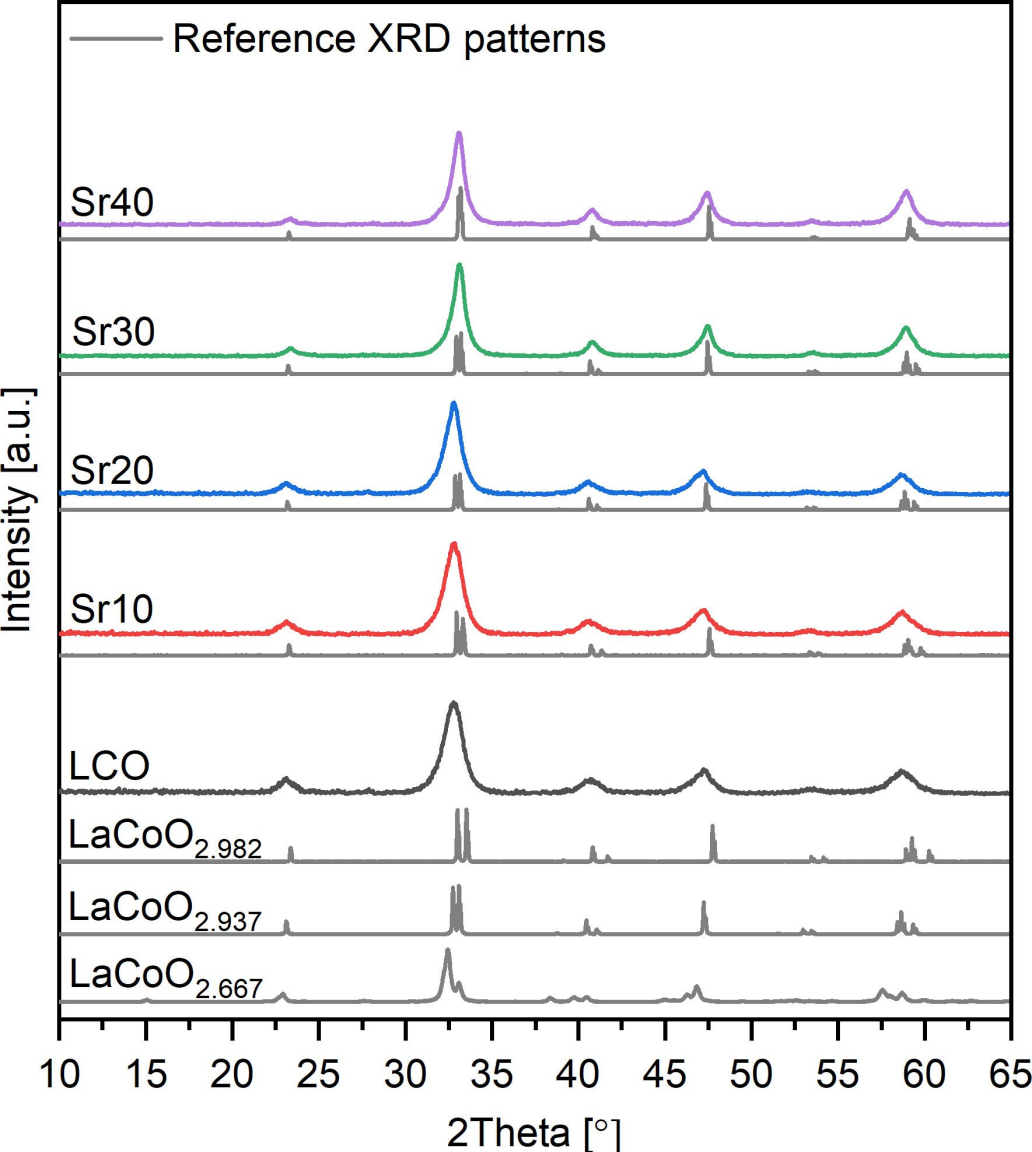
X‐ray diffractograms of the as‐prepared La_1−*x*_Sr_*x*_CoO_3_ perovskites with increasing Sr content. The XRD patterns of LaCoO_2.982_ (ICSD: 153986), LaCoO_2.937_ (ICSD: 153995), LaCoO_2.667_ (ICSD: 86176) are shown as reference patterns to analyze the LCO. La_0.9_Sr_0.1_CoO_3_ (ICSD: 82814), La_0.8_Sr_0.2_CoO_3_ (ICSD: 230621), La_0.7_Sr_0.3_CoO_3_ (ICSD: 164488), and La_0.6_Sr_0.4_CoO_3_ (ICSD: 82817) are plotted as the reference phases of Sr10, Sr20, Sr30 and Sr40.

Figure 1 shows the XRD patterns of the as‐prepared nanoparticles. The XRD pattern of LCO nanoparticles consists of asymmetric and highly broadened reflections. The main diffraction peak positions suggest that LaCoO_2.937_ is the primary phase. The effect of strain was evaluated by Williamson–Hall plots (Figure S1 in the Supporting Information), which indicate that the average microstrain values systematically decrease with increasing Sr amount. Thus, peak shift and asymmetrical broadening in the XRD pattern of LCO can be mainly associated with relatively higher microstrain levels in its nanocrystals, whereas the influence of oxygen deficiency in the perovskite phase is marginal. Therefore, mainly Co^3+^ is present and a low degree of oxygen deficiency can be assumed. On the other hand, with increasing Sr amount, the XRD patterns show a better match with reference stoichiometric patterns and exhibit less broad reflections. This observation can be referred to Co^3+^/Co^4+^ ions present and/or higher concentration of oxygen vacancies, so that charge neutrality is maintained upon substitution of La^3+^ with Sr^2+^. Overall, the phase compositions of La_1−*x*_Sr_*x*_CoO_3_ nanoparticles include perovskite‐structured crystals, and only minor amounts of La(OH)_3_, Co_3_O_4_ and La_2−*x*_Sr_*x*_CoO_4_ were determined as secondary phases (Figure S2).

N_2_ physisorption measurements were carried out to derive the specific surface areas of the La_1−*x*_Sr_*x*_CoO_3_ samples using the BET method (Table 1). The surface areas are high ranging between 81 and 106 m^2^ g^−1^. The corresponding BET particle sizes are in the range from 8.2 to 10.5 nm in good agreement with the count mean diameter determined by TEM measurements amounting to 7.7 to 10.7 nm. In addition, the XRD average crystal sizes are also similar to the particle diameters determined by BET and TEM measurements with only small differences.

**Table 1:** Specific surface areas, particle sizes determined by N_2_ physisorption and TEM, and XRD average crystal sizes calculated from the Williamson‐Hall plots.

Catalyst	Specific surface area [m^2^/g]	BET particle diameter [nm]	TEM count mean diamter [nm]	XRD average crystal size [nm]
LCO	81.6	10.1	7.7	10.5
Sr10	81.8	10.1	8.0	8.3
Sr20	105.7	8.2	8.7	8.2
Sr30	83.6	10.5	10.6	11.4
Sr40	92.3	9.7	10.7	10.1

**Figure 2:**
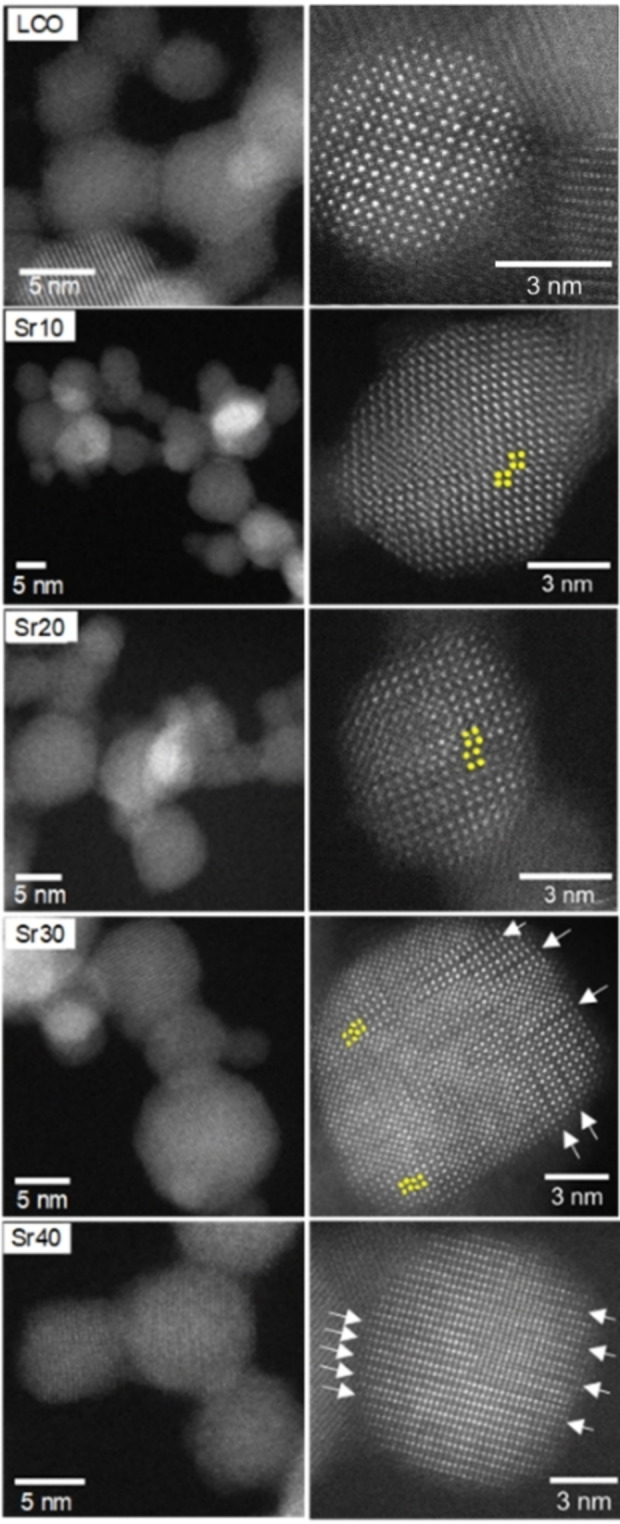
HAADF‐STEM images of the La_1−*x*_Sr_*x*_CoO_3_ samples showing the particle morphology and necking together with the atomic arrangements in the representative nanoparticles. Yellow circles and white arrows both indicate antiphase boundaries. Note: the white arrows can also be related to oxygen‐deficient layers in Sr30 and Sr40 crystals.

Moreover, the degree of sintering between the nanoparticles and the bulk composition of perovskite nanocrystals were investigated by TEM (Figures 2 and S3) and EDX measurements (Figure S4), respectively. In all nanoparticles, particle necking appears without substantial sintering. The EDX elemental mapping of La, Co and Sr shows homogenous distributions throughout the La_1−*x*_Sr_*x*_CoO_3_ nanoparticles. The EDX compositions indicate (La+Sr)/Co atomic ratios near unity and Sr/(La+Sr) ratios very close to the molar fraction of Sr precursor used during spray‐flame synthesis (Table S1). These results confirm the successful incorporation of the desired amount of Sr ions into the perovskite structure.

HAADF‐STEM measurements were performed to examine the nanoparticle structure in detail (Figure 2, right). LCO particles mostly show a single crystalline nanostructure with high crystallinity. Ruddlesden‐Popper‐type antiphase boundaries and Co‐deficient atomic layers are also found in the STEM images of several nanoparticles (Figure S5). These kinds of defects were recently reported in other unsubstituted perovskites,^[29]^ pointing out the complex local structure of perovskite nanocrystals. In Sr10 and S20, most nanocrystals have some degrees of antiphase boundaries. These defects appear more extensively in Sr30 and Sr40. In addition, black stripes are visible in the STEM images of these nanoparticles, which can be associated with oxygen vacancy defects at high Sr contents.[^17^, ^30^] Overall, with increasing Sr content, highly defective crystals appear most likely owing to the mismatch between the ionic radii of La and Sr (*r*_Sr_=1.44 Å vs. *r*_La_=1.36 Å).^[31]^

**Figure 3:**
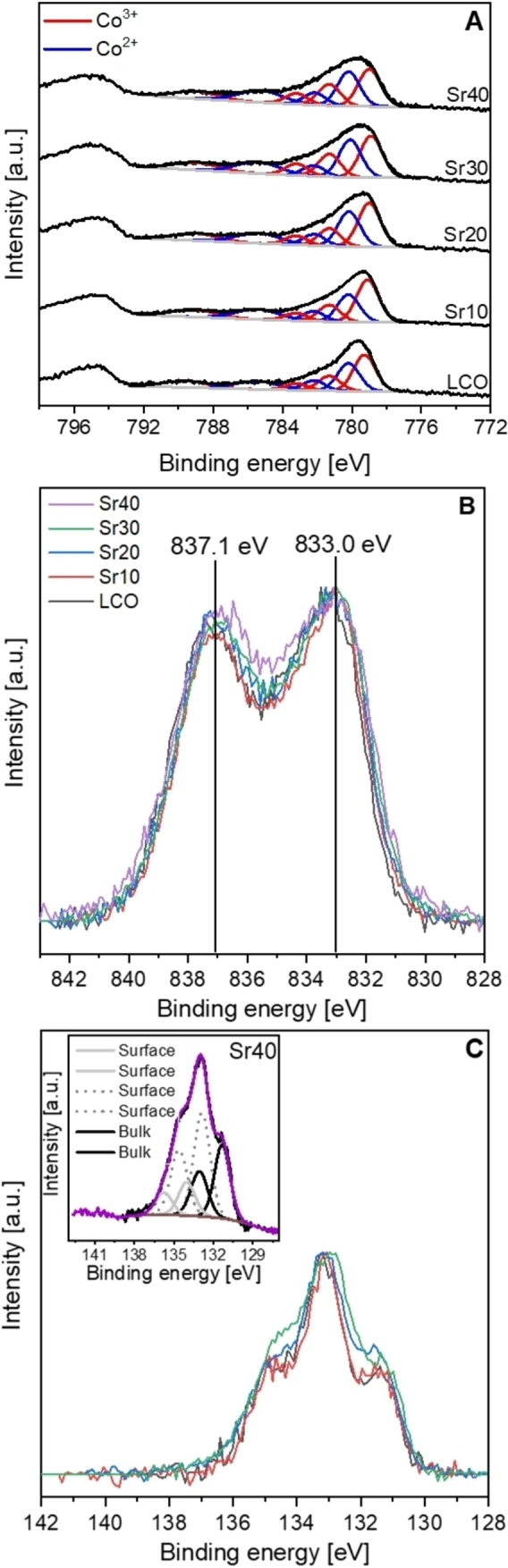
A) Co 2p, B) La 3d, and C) Sr 3d XP spectra of La_1−*x*_Sr_*x*_CoO_3_ nanoparticles. Deconvoluted peaks of Co^3+^ and Co^2+^ are indicated by red and blue peaks, respectively. Deconvoluted peaks of Sr40 are shown in the inset of the Sr 3d region to identify bulk and surface Sr.

In addition to structural analyses, the surface oxidation state of Co and the surface chemical species and composition were investigated by XPS (Figure 3). In all Co 2p spectra, the core‐level and satellite peaks of Co^3+^ appear at 779.1 and 789–789.5 eV, respectively, while the peaks of Co^2+^ are visible at 780.1 and 782.2 eV together with the satellite at 785.4 eV.^[32]^ Compared with the spectrum of LCO, the core‐level peak of Co^2+^ at 780.1 eV and the satellite at 785.4 eV appear with slightly higher intensities for Sr10 and Sr20. Nevertheless, the amounts of Co^2+^ and Co^3+^ in LCO, Sr10, Sr20 are rather similar (atomic ratio of Co^2+^/Co^3+^ is 43/57), so no considerable difference in the Co oxidation state was detected in these samples. The spectra of Sr30 and Sr40 show increasing peak intensities at 780.1 eV relative to those at 779.1 eV, indicating slightly higher concentrations of Co^2+^ ions in these samples.

In the La 3d spectra, the core‐level and satellite peaks are visible at 833 and 837.1 eV with a multiplet splitting of 4.1 eV (Figure 3B). Both the binding energy and multiplet splitting values correspond to the La 3d spectral features of perovskites.^[33]^ For Sr40, the La 3d spectrum exhibits additional low‐intensity peaks between 833 and 837.1 eV, which could be attributed to the signals coming from the secondary phases of La_2−*x*_Sr_*x*_Co_*x*_O_4_ and/or La(OH)_3_.^[34]^

In the Sr 3d spectra, the intensities of bulk Sr peaks are visible at 131.2 and 133 eV, whereas those of surface Sr appear between 138–132 eV (Figure 3C, inset). These spectra show high similarity to the previously reported Sr 3d spectra of La_1−*x*_Sr_*x*_CoO_3_ perovskites, showing high contents of surface Sr components, which can originate from Sr‐terminated perovskite surfaces and/or the surface of secondary phases.[^32a^, ^35^] In particular, the Sr 3d spectra of Sr10 and Sr20 appear rather similar, while those of Sr30 and Sr40 show relatively larger contents of lattice Sr and surface Sr species, respectively. For Sr30, higher intensity of lattice Sr with respect to surface Sr can be attributed to the lower content of secondary phases. On the contrary, the enhanced intensity of surface Sr in Sr40 catalysts can be related to the relatively higher content of La_2−*x*_Sr_*x*_CoO_4_ (Figure S2). Overall, most Sr 3d intensities can be related to Sr perovskite components, and the minor changes in the relative proportions of the lattice and surface components can be explained by the secondary phases.

The surface chemical groups in the C 1s and O 1s spectra were also analyzed, indicating slightly higher concentrations of carbonates and carboxylates on Sr20 (Figures S6 and S7). ATR‐FTIR spectra identified higher amounts of bulk carbon species with increasing Sr amount. The O 1s spectra show an increase in the O_2_^2−^/O^−^ peak intensity for Sr30 and Sr40, which can be related to oxygen vacancies in the perovskite materials^[36]^ as well as oxygen species of Co_3_O_4_.^[37]^

In addition, the surface concentrations of carbon and oxygen in La_1−*x*_Sr_*x*_CoO_3_ catalyst were determined by the quantitative analysis of the regional XP spectra (Table 2). LCO, Sr10, Sr30 and Sr40 show surface carbon concentrations of about 20 at. %, while Sr20 has a relatively lower carbon concentration of about 15 at. %. Considering the surface carbon concentration and carbon‐bound oxygen O_carbon_, the surface of Sr20 has the lowest content of carbonaceous combustion residuals. Moreover, since lattice oxygen and O_2_^2−^/O^−^ are mainly involved in catalytic oxidation,^[38]^ their overall oxygen concentrations were calculated by subtracting O_carbon_ from the total oxygen concentration (O_total_−O_carbon_). This results in a concentration lower than 42 at. % for LCO, Sr10, Sr30 and Sr40, while Sr20 has a O_total_−O_carbon_ concentration of about 47 at. %. These results suggest that Sr20 contains a higher concentration of catalytically active oxygen species compared with the other catalysts, owing to the lower concentration of carbon residuals at the surface.

**Table 2:** Relative abundance [at. %] of carbon, carbon‐bound oxygen (O_carbon_) and lattice and surface adsorbed oxygen in La_1−*x*_Sr_*x*_CoO_3_.

Catalyst	C	O_carbon_	O_total_−O_carbon_
LCO	19.7	11.4	40.3
Sr10	20.1	9.0	41.8
Sr20	14.5	7.9	46.9
Sr30	19.9	10.1	39.2
Sr40	19.4	11.4	41.0

Information on the Co spin states and the magnetic properties of the La_1−*x*_Sr_*x*_CoO_3_ materials was obtained by X‐band cw EPR at 10 K and temperature‐dependent DC SQUID magnetization measurements. The EPR spectra (Figure S8) of all samples exhibit strong high spin (*S*=3/2) Co^2+^ signals.^[39]^ The LCO sample shows an EPR spectrum characteristic for Co^2+^ in octahedral coordination. Sr‐containing samples display higher Co^2+^ signal intensity and changes in the EPR line shape, suggesting changes in the Co^2+^ coordination environment upon Sr doping.^[40]^ The Co^2+^ content increases by approximately 12‐fold for Sr40 and by fourfold for Sr10 with respect to LCO. The noticeably higher EPR signal intensities of Sr40 compared with samples with lower Sr doping levels may originate from an increase of high‐spin Co^2+^ EPR signals of the secondary Co_3_O_4_ and La(Sr)_2_CoO_4_ phases in Sr40 (Figure S2). Neither Co^4+^ nor signals from paramagnetic oxygen vacancies are observed in the EPR spectra. Note that octahedrally coordinated Co^3+^ in *S*>0 states of cobalt perovskites cannot be detected by conventional EPR due to its large spin couplings.^[41]^

Temperature‐dependent DC magnetization measurements on field‐cooled (FC) and zero‐field‐cooled (ZFC) samples reveal different magnetic phases depending on the doping level (Figure S9). Sr20, Sr30 and Sr40 exhibit clear evidence for transitions from a ferromagnetic to a paramagnetic phase upon increasing the temperature, which disappears at lower doping levels. The resulting Sr doping‐dependent magnetic phase diagram of La_1−*x*_Sr_*x*_CoO_3_ (Figure S10) is in close resemblance with data obtained on similar materials.^[42]^ This accordance provides evidence that despite the significant amount of Co^2+^ in the sample the magnetic properties are as expected for this class of materials.

**Figure 4:**
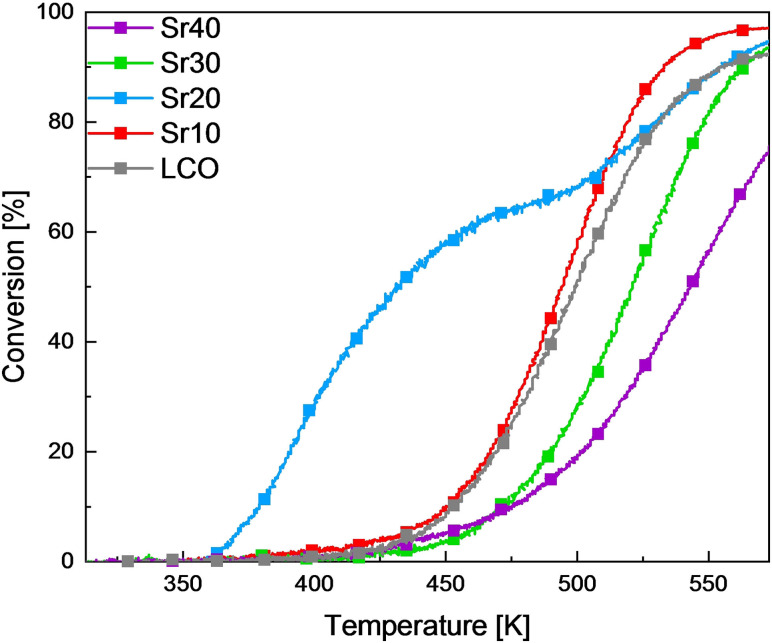
Influence of Sr amount on degrees of conversion during gas‐phase propan‐2‐ol oxidation over the La_1−*x*_Sr_*x*_CoO_3_ catalysts. Reaction conditions: 10 mg catalyst, 0.18 % propan‐2‐ol/0.18 % O_2_/He feed, 0.5 K min^−1^, 313–573 K.

Gas‐phase propan‐2‐ol oxidation is a suitable method to investigate mechanistic aspects by probing three different reaction routes: oxidative dehydrogenation forming acetone and water, dehydrogenation forming acetone and H_2_, and dehydration forming propene and water. Total oxidation of propan‐2‐ol or its reaction intermediates is observed at high temperatures. The dehydrogenation yielding acetone requires moderate acidic and strong basic sites, while dehydration yielding propene takes place at strong acidic and weak basic sites.^[43]^ Nevertheless, in metal oxide catalysis, the redox properties often predominate over the acid‐base properties determining the oxidation activity. Consequently, propan‐2‐ol is also a probe reaction of the redox properties. Figure 4 shows gas‐phase propan‐2‐ol oxidation over La_1−*x*_Sr_*x*_CoO_3_ nanoparticles. The high degrees of conversion and yields of acetone identify the La_1−*x*_Sr_*x*_CoO_3_ nanoparticles as active and selective catalysts for propan‐2‐ol oxidation (Table 3). The samples containing 0, 10, 20 and 30 at. % Sr show similar high‐temperature activity of *X*>90 % at 573 K, whereas propan‐2‐ol oxidation over Sr40 only reaches a maximum conversion of 75 %. By substituting La^3+^ with Sr^2+^ up to 20 % in the A‐site of LaCoO_3_, the catalytic activity increased, but it decreased with further substitution. The same trend is observed for the acetone yield being the highest over Sr20 and Sr30 with 86 %, whereas the CO_2_ yield formed by total oxidation gradually decreases with increasing Sr substitution. No propene was formed. Sr20 is the only sample which also shows a strong low‐temperature activity promoting propan‐2‐ol oxidation already at 360 K. The high conversion and acetone yields over La_1−*x*_Sr_*x*_CoO_3_ nanoparticles demonstrate superior catalytic redox activity.

**Table 3:** Yields of gas‐phase propan‐2‐ol oxidation over the La_1−*x*_Sr_*x*_CoO_3_ samples at 573 K.

Sample	Yield [%]		
	Acetone	CO_2_	Propene
LCO	82	11	0
Sr10	84	13	0
Sr20	86	9	0
Sr30	86	8	0
Sr40	69	5	0

In summary, the STEM images revealed defective crystal structures with increasing Sr amount and the Co 2p XP spectra identified increasing Co^2+^ concentrations relative to Co^3+^, implying an oxygen‐deficient surface composition of the perovskite nanoparticles. In accordance with XPS, EPR measurements revealed increasing concentrations of high‐spin Co^2+^ species with increasing Sr doping and excluded the presence of Co^4+^. The O 1s XP spectra detected increasing O_2_^2−^/O^−^ intensities for higher Sr amounts, indicating the formation of oxygen vacancies. Thus, the incorporation of Sr into the A site of the perovskite structure does not result in the formation of Co^4+^ species, but leads to the formation of oxygen vacancies to maintain charge neutrality.

### Influence of Sr content on cyclohexene oxidation with O_2_

**Figure 5:**
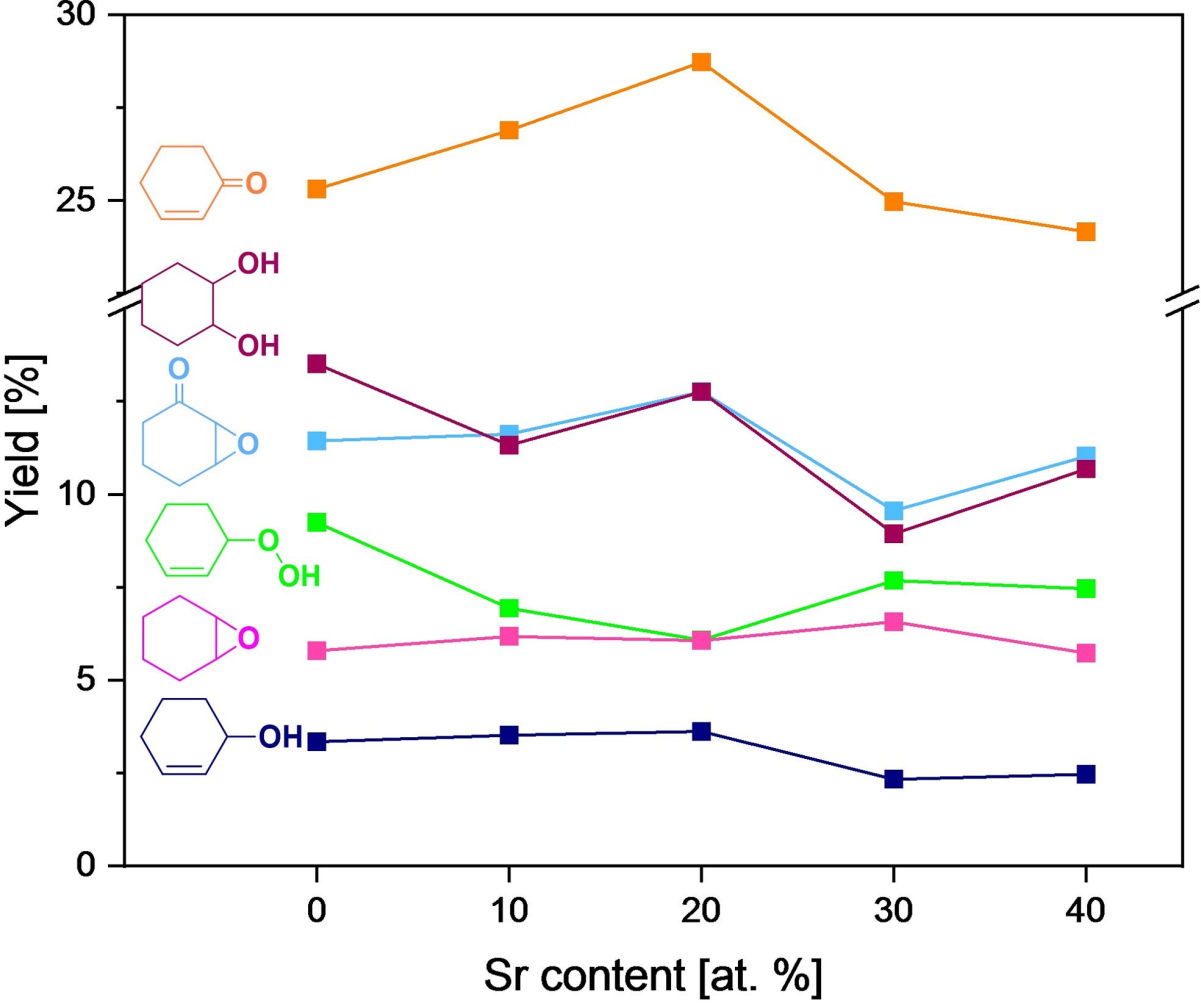
Cyclohexene oxidation yields over different La_1−*x*_Sr_*x*_CoO_3_ perovskites containing 0, 10, 20, 30 and 40 at. % Sr under standard conditions after 6 h.

Perovskite nanoparticles show promising activities in liquid‐phase oxidation reactions.[^26b^, ^44^] The insertion of Sr into LaCoO_3_ might enhance the catalytic activity because of the formation of oxygen vacancies.^[45]^ To study the effect of Sr doping, cyclohexene oxidation with molecular O_2_ was carried out over La_1−*x*_Sr_*x*_CoO_3_ nanoparticles in acetonitrile at 80 °C (Figure S11). The initial reaction rate determined within the first hour is the highest over Sr20, leading to 26.7 % cyclohexene conversion, whereas higher and lower Sr amounts promote cyclohexene conversion in a less pronounced way. Meanwhile, the hydroperoxide selectivity is strikingly lowered by 15 % over Sr20 compared with LCO or Sr40. The strong ability of Sr20 to decompose the hydroperoxide thus leads to higher product yields, mainly yielding the ketone cyclohex‐2‐ene‐1‐one. The selectivity to other reaction products such as 7‐oxabicyclo[4.1.0]‐heptane‐2‐one and cyclohexane‐1,2‐diol is similar over all investigated catalysts in the range of 10 to 15 %. The stable and low selectivity of 4 % to the epoxidation product cyclohexene oxide indicates a direct consecutive reaction to the diol. cyclohex‐2‐ene‐1‐ol selectivity decreases over time, indicating its consecutive oxidation to the corresponding ketone (Scheme 1). The comparison of the product yield after 6 h shows a similar trend regarding the influence of Sr doping (Figure 5). Because cyclohexene oxidation is able to undergo two different reaction pathways, the oxidation products are correspondingly divided into allylic and epoxidation products (Table S2). cyclohex‐2‐ene‐1‐one, cyclohex‐2‐ene‐1‐ol and 7‐oxabicyclo[4.1.0]heptan‐2‐one are allylic products, while cyclohexene oxide and cyclohexane‐1,2‐diol are products of the epoxidation pathway. An increase of the Sr amount up to 20 at. % leads to an increase in allylic yield up to 40.8 %, whereas higher Sr amounts again decrease the yield by 6.4 and 3.0 % over Sr30 and Sr40, respectively. The main allylic product is the ketone with a yield of 28.8 %, followed by 7‐oxabicyclo[4.1.0]heptan‐2‐one with 12.8 %. Both reaction products show a dependence on the incorporated Sr amount. However, the yield of cyclohex‐2‐ene‐1‐ol is stable at a low level of 3.6 % independent of the Sr amount. The increase in allylic selectivity over Sr20 can be explained by the faster hydroperoxide decomposition: the hydroperoxide yield is decreased by 3.9 % over Sr20 compared with LCO (11.6 %). The yield of epoxidation products is not strongly affected by Sr doping. Within the epoxidation products, cyclohexane‐1,2‐diol is the main product with a yield of 20.0 % over Sr20, whereas cyclohexene oxide is formed with 6.0 % yield over all investigated catalysts independent of the Sr amount.

Comparing the catalytic properties of herein synthesized perovskites with a commercial analogue shows the superior catalytic activity and selectivity of the spray‐flame‐made nanoparticles with high surface areas (Figure S12). A commercial La_0.8_Sr_0.2_CoO_3_ catalyst exhibits only 46.5 % cyclohexene conversion, whereas our Sr20 sample achieves a high conversion of 78.9 %. The comparison also clarifies the outstanding hydroperoxide decomposition ability of Sr20, shifting the selectivity to cyclohex‐2‐ene‐1‐one as the main product. In contrast, the commercial sample shows a high selectivity to the hydroperoxide intermediate, resulting in low product yields. The low catalytic activity of the commercial sample can be ascribed to its significantly lower surface area of 10 m^2^ g^−1^ and a lower phase purity. Correlating the specific surface areas and particle sizes of the La_1−*x*_Sr_*x*_CoO_3_ catalysts with their catalytic activities does not show a clear dependence on cyclohexene conversion, indicating that the slightly larger surface area and smaller particle size of the Sr20 catalyst do not play a crucial role in its higher catalytic activity in terms of hydroperoxide decomposition (Figure S13). Moreover, XRD patterns recorded after cyclohexene oxidation do not indicate any changes in phase purity or crystallinity (Figure S14).

Overall, the experiments show that the incorporation of 20 at. % Sr into LaCoO_3_ nanoparticles results in increased cyclohexene conversion and ketone yield. This result can be attributed to the increasing concentration of oxygen vacancies with increasing Sr amount, as indicated by HAADF‐STEM (Figure 2, right) and XPS (Figure 3), which is beneficial for selective oxidation. The higher concentration of catalytically active oxygen species in Sr20 determined by XPS (Table 2) may also play a major role in its superior activity, which is also underlined by its outstanding activity in gas‐phase 2‐propanol oxidation. However, a further increase of the Sr content to 30 and 40 at. % is detrimental to catalytic activity. This is probably due to the increased surface concentration of Co^2+^ with respect to Co^3+^ indicated by the Co 2p and O 1s XP spectra and the EPR spectra (Figures 3, S7, and S8), as Co^3+^ cations are the catalytically active species for selective oxidation.^[46]^ However, Co^2+^ cations are known to be the key factor for the decomposition of peroxides.^[47]^ Therefore, Sr20 is identified to be the optimum catalyst having a balanced amount of oxygen vacancies and Co^2+^. In this way, the catalyst has a sufficient amount of Co^3+^ and oxygen vacancies to activate and oxidize the cyclohexene molecule to the hydroperoxide intermediate, but also a certain amount of Co^2+^ cations that can rapidly decompose the cyclohex‐2‐ene‐1‐hydroperoxide to the desired products.

### Kinetic investigations over Sr20

**Figure 6:**
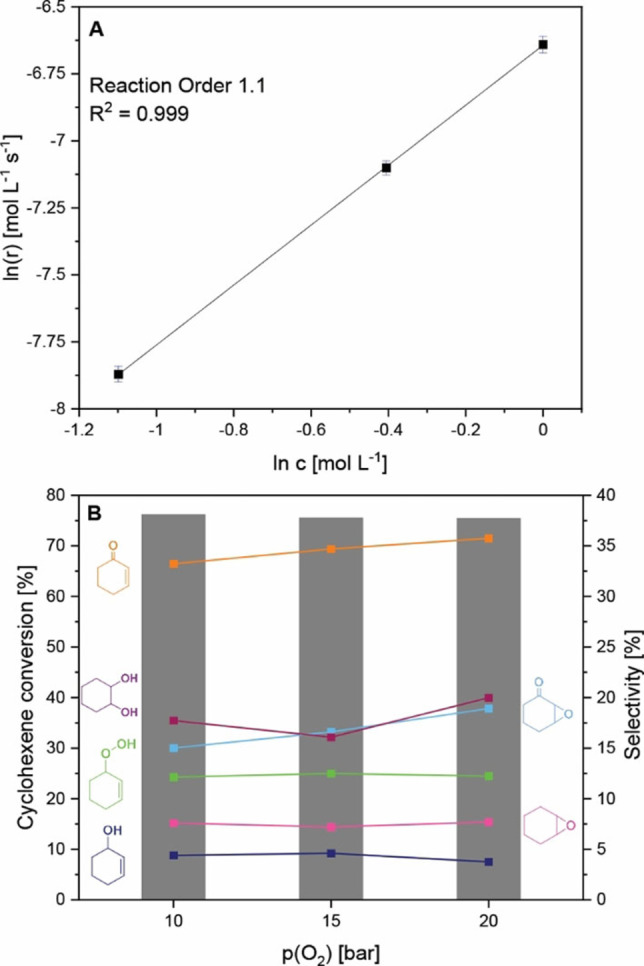
A) Linearized plot of the reaction rate over Sr20 as a function of the initial cyclohexene concentration. B) Effect of O_2_ pressure on cyclohexene conversion and product selectivity over Sr20.

The catalyst Sr20 was chosen for further kinetic investigations because of its superior catalytic activity towards cyclohexene oxidation compared with LaCoO_3_. The kinetics over LaCoO_3_ have already been discussed previously.^[9]^ The increase in cyclohexene conversion as a function of initial cyclohexene concentration is shown in Figure S15. The selectivities to the main products cyclohex‐2‐ene‐1‐one, 7‐oxabicyclo[4.1.0]heptan‐2‐one and cyclohexane‐1,2‐diol are stable for all initial cyclohexene concentrations. Furthermore, no changes in phase purity and crystallinity were observed in the XRD patterns of the fresh and spent catalysts (Figure S14D). The linearized plot of the reaction rate as a function of initial cyclohexene concentration reveals first‐order reaction kinetics with high accuracy (Figure 6A). Thus, with increasing initial cyclohexene concentration, cyclohexene oxidation increases too, and meanwhile, a strong increase in hydroperoxide selectivity is observed. This observation indicates the hydroperoxide decomposition to be the rate‐determining step of product formation as the decomposition cannot keep up with the rate of cyclohexene oxidation. As acetonitrile is a strong coordinating ligand, ATR‐IR spectra were recorded to study the competing adsorption of acetonitrile and cyclohexene and to exclude the cyclohexene adsorption on the catalyst surface being the rate‐determining step in cyclohexene oxidation. Figure S16 shows the time‐dependent difference spectra of a mixture of 0.67 mol L^−1^ cyclohexene dissolved in acetonitrile. Already after 5 min, the characteristic cyclohexene bands are prominent and acetonitrile bands vanish, confirming that cyclohexene is able to adsorb on the perovskite surface.

Cyclohexene conversion and product selectivity as a function of applied O_2_ pressure are shown in Figures 6B and S15. Independent of the O_2_ pressure, cyclohexene conversion is constant. Slight changes in product selectivity are observed resulting from a slower hydroperoxide decomposition with increasing O_2_ pressure. Control experiments show no dependence on the stirring speed above 600 rpm, so that O_2_ mass transport limitation can be excluded (Figure S18). Unchanged XRD patterns (Figure S15D) of the spent catalysts indicate the high stability of the Sr20 nanoparticles towards high pressure. These results confirm that cyclohexene oxidation is not limited by the applied O_2_ pressure revealing a zero‐order dependence on O_2_ pressure. A control experiment in the absence of O_2_ showed no cyclohexene conversion after 6 h of reaction, indicating that O_2_ is indispensable for cyclohexene oxidation and that surface or lattice oxygen from the perovskite oxide is not involved.

**Figure 7:**
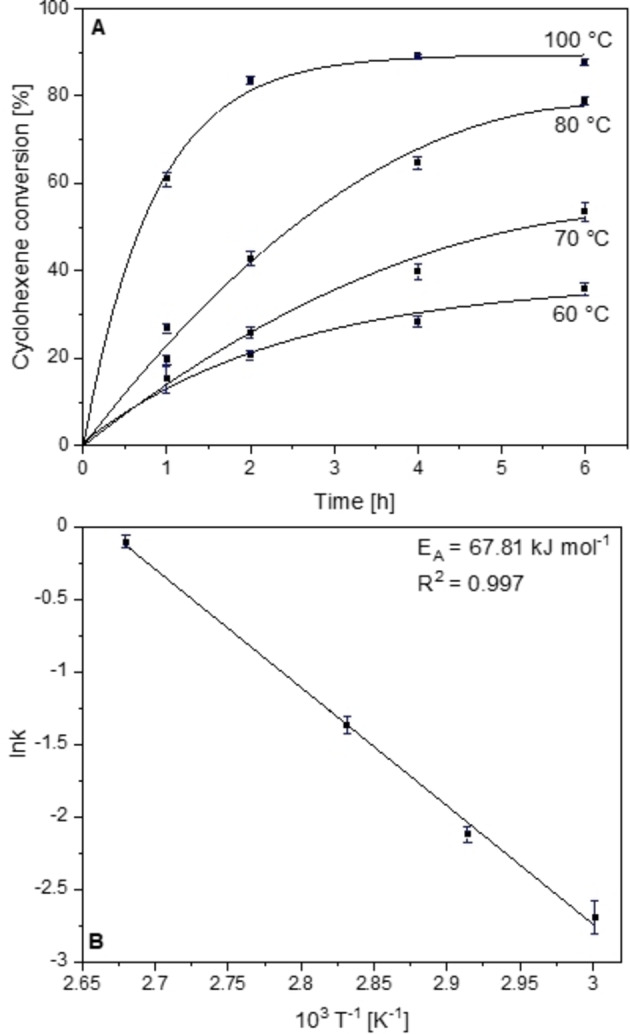
A) Cyclohexene conversion over Sr20 as a function of time and B) Arrhenius analysis based on first‐order reaction kinetics.

The continuous increase in cyclohexene conversion as a function of temperature is presented in Figures 7A and S16. Cyclohexene conversion is increased from 35.8 % at 60 °C to 87.8 % at 100 °C after a reaction time of 6 h and otherwise standard conditions. The increase in temperature led to different product distributions, because higher temperatures favor hydroperoxide decomposition: the hydroperoxide selectivity decreases from 59.8 % at 60 °C to 0.0 % at 100 °C after 6 h, suggesting that the rate of hydroperoxide decomposition strongly depends on temperature. Hydroperoxide decomposition mainly results in a selectivity increase of cyclohex‐2‐ene‐1‐one, but also 7‐oxabicyclo[4.1.0]heptan‐2‐one, cyclohexane‐1,2‐diol and cyclohexene oxide are produced in higher amount. With faster hydroperoxide decomposition, both allylic and epoxidation selectivities increase, indicating that both reaction pathways include the hydroperoxide as intermediate (Scheme 1). Only the selectivity to cyclohex‐2‐ene‐1‐ol decreases with increasing temperature suggesting that the overoxidation of the alcohol to the ketone is favoured at higher temperatures.

The linearized first‐order reaction plot is well suited for linear regression (*R* ^2^>0.94), indicating again that cyclohexene oxidation over Sr20 follows first‐order reaction kinetics (Figure S19). The Arrhenius analysis results in a plot with high accuracy (*R* ^2^=0.997) revealing an apparent activation energy of 67.8 kJ mol^−1^ (Figure 7B).

**Figure 8:**
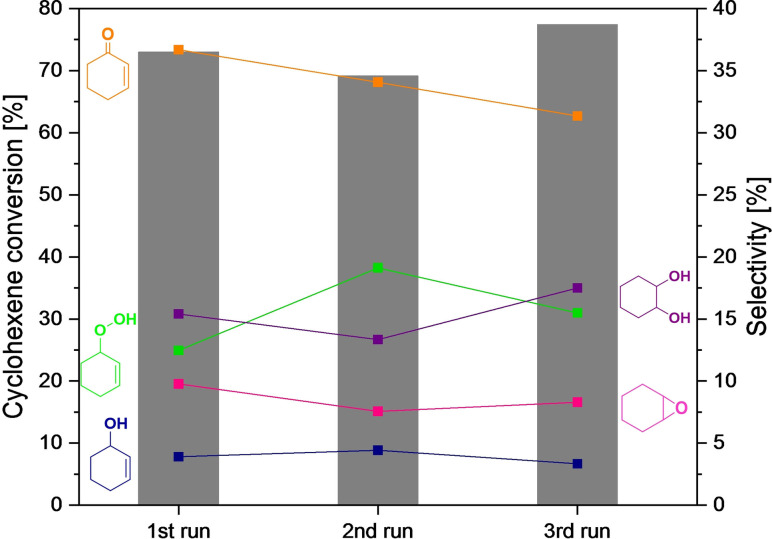
Reusability test for cyclohexene oxidation over Sr20: cyclohexene conversion and product selectivity in three consecutive oxidation runs.

The reusability test of cyclohexene oxidation over Sr20 is shown in Figure 8. After three consecutive reaction cycles, cyclohexene conversion and product selectivity still remained comparable to the values of the first run. Only small changes in hydroperoxide decomposition were observed resulting in slightly lower product yields. The XRD patterns of the recycled catalysts indicate that the main characteristics were preserved during the recycle tests (Figure S20A). STEM images of the fresh and the spent catalyst after three runs show that changes in particle size or morphology did not occur (Figure S20). EDX mapping of the fresh catalyst shows an enrichment of Co on the surface of the particles with otherwise homogeneous distributions of all elements (Figure S20 B and C). After the reusability test, Co is not the predominant metal on the surface anymore, but some small aggregations of La are detected (Figure S20 D and E). Nevertheless, these changes in metal distribution on the catalyst surface do not immediately influence the catalytic activity of the investigated Sr20 catalyst, which shows high stability in particle size and morphology as well as crystallinity and phase purity for cyclohexene oxidation with molecular O_2_.

### EPR spin trap studies

**Figure 9:**
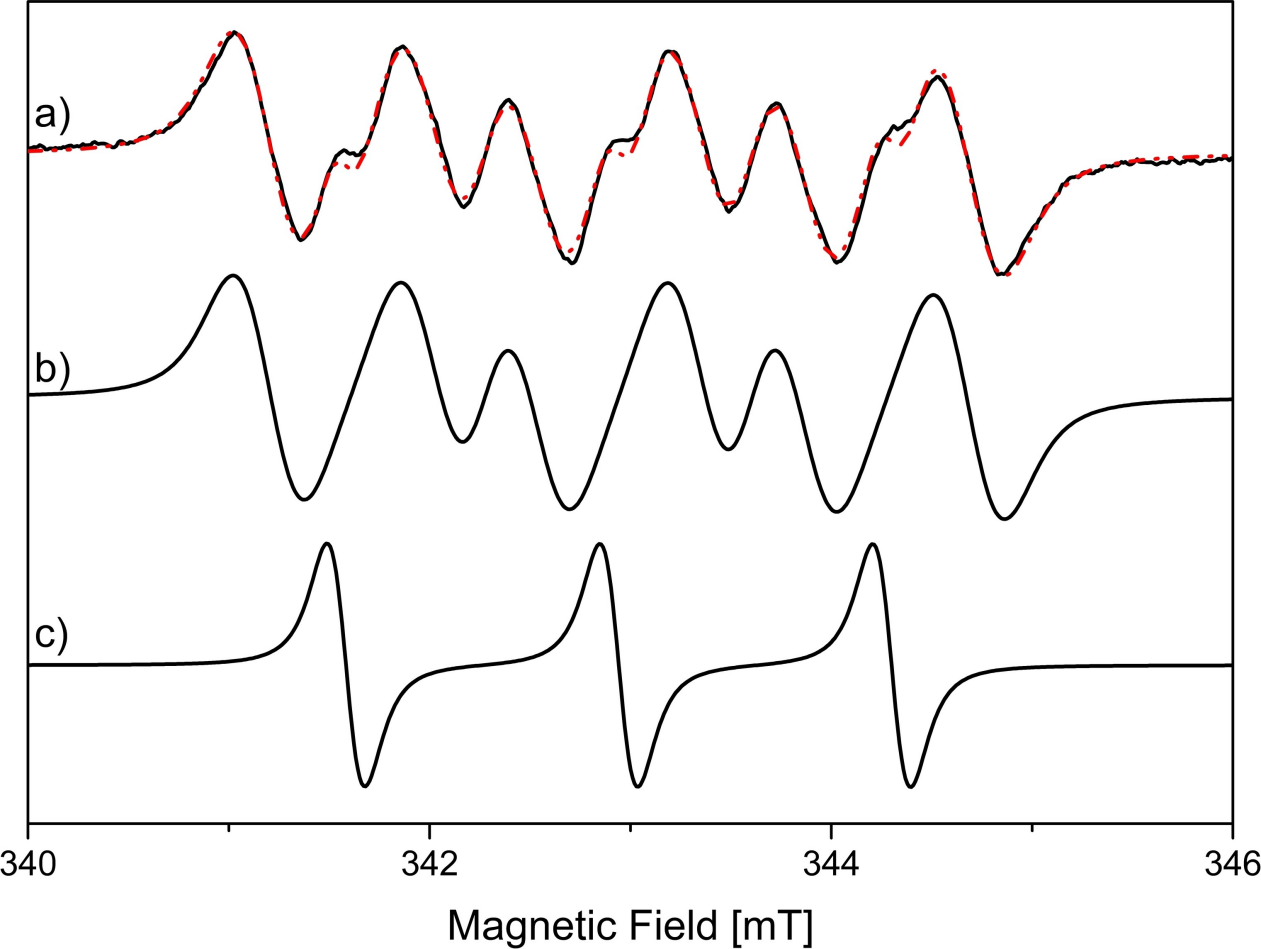
a) EPR spectra obtained at 295 K (black solid line) and corresponding simulation (red dashed line). Deconvolution of the simulated spectrum is shown: b) DMPO/^.^OC_6_H_9_ and c) di‐*tert*‐butyl‐nitroxide derivative. The spin Hamiltonian parameters used for simulation are: 75 % DMPO/^.^OC_6_H_9_ (*g*=2.006, *a*_N_=1.33 mT, *a*_Hα_=0.83 mT, *a*_Hβ_=0.14 mT and Voigtian linewidth broadening comprising 0.234 mT Gaussian and 0.325 mT Lorentzian) and 25 % di‐*tert*‐butyl‐nitroxide derivative (*g*=2.006, *a*_N_=1.36 mT and Voigtian linewidth broadening comprising 0.126 mT Gaussian and 0.220 mT Lorentzian).

EPR spin trap experiments were performed to identify possible radical pathways during cyclohexene oxidation. Spin traps, like the herein used 5,5‐dimethyl‐1‐pyrroline *N*‐oxide (DMPO), are molecules that form paramagnetic spin adducts upon reaction with reactive oxygen species and/or carbon‐centered radicals.^[48]^ These spin adducts show characteristic EPR spectra, determined by hyperfine coupling constants (*a*_N_ and *a*_H_) of the unpaired electron spin and nuclei on the DMPO moiety. Cyclohexene oxidation over Sr40 was carried out to investigate the present radicals. Whereas control samples without DMPO added to the solution showed no EPR signal, samples prepared with DMPO exhibited the characteristic EPR of DMPO trapped radicals. Figure 9 shows the room temperature EPR spectrum obtained after 20 min in presence of DMPO. Simulation of the spectrum revealed a superposition of two spin adducts containing 75 % alkoxyl radical adduct and 25 % di‐*tert*‐butyl‐nitroxide derivative.^[49]^ The alkoxyl radical is assigned to a cyclohexenyl alkoxyl radical (^.^OC_6_H_9_) formed upon oxidation of ^.^C_6_H_9_ during cyclohexene oxidation. This cyclohexenyl alkoxyl radical has been found in literature for manganese porphyrin‐catalyzed oxidation of diphenylmethane in presence of cyclohexene using PBN as a spin trap.^[50]^ The presence of the di‐*tert*‐butyl‐nitroxide derivative is probably due to decomposition of DMPO and is therefore not further considered. In summary, EPR spin trap experiments identified cyclohexene oxidation to proceed according to a radical pathway including the cyclohexenyl alkoxyl radical.

### Time‐resolved in situ ATR‐IR spectroscopy

Cyclohexene oxidation follows a complex reaction network (Scheme 1), enabling the formation of various reaction products. To verify the different reaction pathways of cyclohexene oxidation, in situ ATR‐IR spectroscopy was employed. This method enables the time‐resolved investigation of cyclohexene consumption and product formation.

Prior to the in situ measurement, ATR‐IR spectra of reference samples in acetonitrile were recorded (Figure S21) to assign the characteristic bands to the reactant and the oxidation products, which are summarized in Table S3.^[51]^ The identification of reaction products and especially of the cyclohex‐2‐ene‐1‐hydroperoxide intermediate was discussed recently.^[51]^

**Figure 10:**
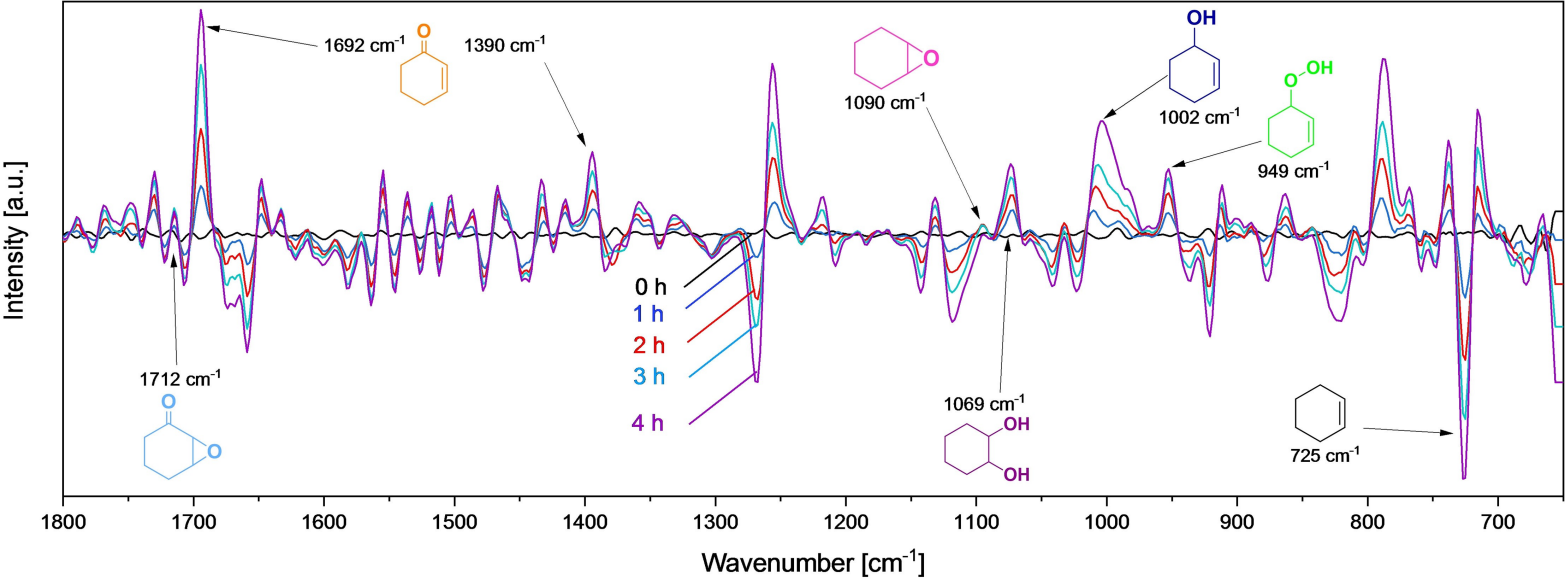
In situ ATR‐IR spectra for the oxidation of cyclohexene over Sr40 catalyst after 0, 1, 2, 3 and 4 h in the range of 1800 to 650 cm^−1^ after background subtraction. The spectrum for background subtraction is taken from a mixture of acetonitrile, *o*‐dichlorobenzene as internal standard for GC analysis, cyclohexene and Sr40 catalyst under N_2_ at 80 C. The first derivatives of obtained raw spectra are shown.

The reaction progress of cyclohexene oxidation was continuously monitored by recording IR spectra every 2 min. Figure 10 shows the first‐derivative spectra after admitting a solution of cyclohexene in acetonitrile containing Sr40 nanoparticles as the catalyst (Figure S22). Previously, the solution had been heated to 80 °C under inert N_2_ atmosphere before pressurizing with molecular O_2_ and initiating the actual cyclohexene oxidation reaction (0 h). The recorded spectrum at 0 h is used for background subtraction. Similar to the spectral change over time in Figure 10, the qualitative trend of the C=C bending band at 720 cm^−1^ gradually decreases, representing the consumption of cyclohexene. Cyclohexene conversion and product selectivity were simultaneously determined by offline GC analysis (Figure S23). After 4 h, roughly 50 % of cyclohexene has been converted into its reaction products. All reaction products quantified by GC analysis can also be observed spectroscopically. The strong increase in the intensity of the C=O stretching vibration at 1690 cm^−1^ is due to the formation of cyclohex‐2‐ene‐1‐one, which is the main oxidation product (11.7 % yield) besides the hydroperoxide intermediate (10.2 %), the formation of which is represented by the vibration at 949 cm^−1^. Note that the characteristic vibration of the hydroperoxide is very weak so that peak intensities do not represent quantitative yields. In addition, the intensity of the stretching vibration at 1002 cm^−1^ is significantly increasing, demonstrating the formation of cyclohex‐2‐ene‐1‐ol with a yield of 3.4 % determined by offline GC analysis. Also, the formation of 7‐oxabicyclo[4.1.0]heptan‐2‐one (3.3 % yield) can be monitored by the increase in band intensity at 1713 cm^−1^ so that the formation of all reaction products assigned to the allylic pathway of the cyclohexene oxidation can be traced by in situ ATR‐IR spectroscopy. The selectivity to cyclohexene oxide as an epoxidation product is low, resulting in a yield of only 1.8 %, which is underlined by the low intensities of the assigned band for the alkoxy C−O stretching vibration at 1005 cm^−1^. The C−O stretching band at 1066 cm^−1^ assigned to cyclohexane‐1,2‐diol shows a significantly stronger increase in intensity. Also, GC analysis reveals a higher yield of cyclohexane‐1,2‐diol of 3.0 % as a consecutive product of cyclohexene oxide. Thus, it can be concluded that the hydrolysis of cyclohexene oxide to the diol occurs rapidly (Scheme 1).

## Conclusion

La_1−*x*_Sr_*x*_CoO_3_ nanoparticles with different amounts of Sr were prepared by spray‐flame synthesis and fully characterized by XRD, N_2_ physisorption, TEM, HAADF‐STEM, FTIR, XPS, SQUID magnetometry, and EPR. The La_1−*x*_Sr_*x*_CoO_3_ nanoparticles were found to have small particle sizes between 7.7 and 10.7 nm and large specific surface areas between 81.6 and 105.7 m^2^ g^−1^. With increasing amounts of Sr, an increasing concentration of surface oxygen vacancies was detected. Nevertheless, enrichment of catalytically less active Co^2+^ on the surface and in the bulk was observed by XPS and EPR, respectively. Furthermore, EPR measurements excluded the presence of Co^4+^ ions. SQUID magnetometry exhibits a transformation from a ferromagnetic to a paramagnetic phase for doping levels with *x*≥0.2.

For cyclohexene oxidation, Sr20 led to the highest conversion and selectivity, whereas higher or lower Sr amounts exhibited less pronounced catalytic activity. This can be attributed to the balanced amount of oxygen vacancies and Co^2+^ cations in Sr20 caused by the substitution of La^3+^ with Sr^2+^. Whereas oxygen vacancies and Co^3+^ cations are known to have a superior activity towards hydrocarbon activation, Co^2+^ cations are less active for cyclohexene oxidation, but they are indispensable for the decomposition of the central hydroperoxide intermediate. Furthermore, the higher activity of Sr20 may be ascribed to its higher concentration of catalytically active oxygen species. Kinetic investigations revealed a first‐order and a zero‐order dependence on cyclohexene concentration and O_2_ pressure, respectively. An apparent activation energy of 68 kJ mol^−1^ was determined by Arrhenius analysis for cyclohexene oxidation over Sr20. Reusability tests showed stable cyclohexene conversion and product selectivity within three consecutive reaction runs. This observation is supported by the unchanged diffractograms, particle sizes and morphologies of the spent catalysts. Still, a change in the surface enrichment from Co to La was observed according to EDX elemental mapping. Spin trap EPR revealed that cyclohexene oxidation proceeds via a cyclohexenyl alkoxyl radical (^.^OC_6_H_9_) formed upon oxidation of ^.^C_6_H_9_. In situ ATR‐IR spectroscopy was employed to monitor reaction progress; this confirmed the complex reaction network with cyclohex‐2‐ene‐1‐hydroperoxide as key intermediate.

## Conflict of interest

The authors declare no conflict of interest.

## Supporting information

As a service to our authors and readers, this journal provides supporting information supplied by the authors. Such materials are peer reviewed and may be re‐organized for online delivery, but are not copy‐edited or typeset. Technical support issues arising from supporting information (other than missing files) should be addressed to the authors.

Supporting Information
