# Supplementary material for: Liquid‐Phase Cyclohexene Oxidation with O2 over Spray‐Flame‐Synthesized La1−x Sr x CoO3 Perovskite Nanoparticles
Source: Chemistry. 2021 Oct 14;27(68):16912–23. doi: 10.1002/chem.202103381 (PMC9293428; doi:10.1002/chem.202103381)
Supplement: Supplementary file 1 — Supporting Information [file CHEM-27-16912-s001.pdf]

Chemistry–A European Journal

Supporting Information

# Liquid-Phase Cyclohexene Oxidation with $O_2$ over Spray-Flame-Synthesized $La_{1-x}Sr_xCoO_3$ Perovskite Nanoparticles

Julia Büker, Baris Alkan, Sonia Chabbra, Nikolai Kochetov, Tobias Falk, Alexander Schnegg, Christof Schulz, Hartmut Wiggers,* Martin Muhler, and Baoxiang Peng*

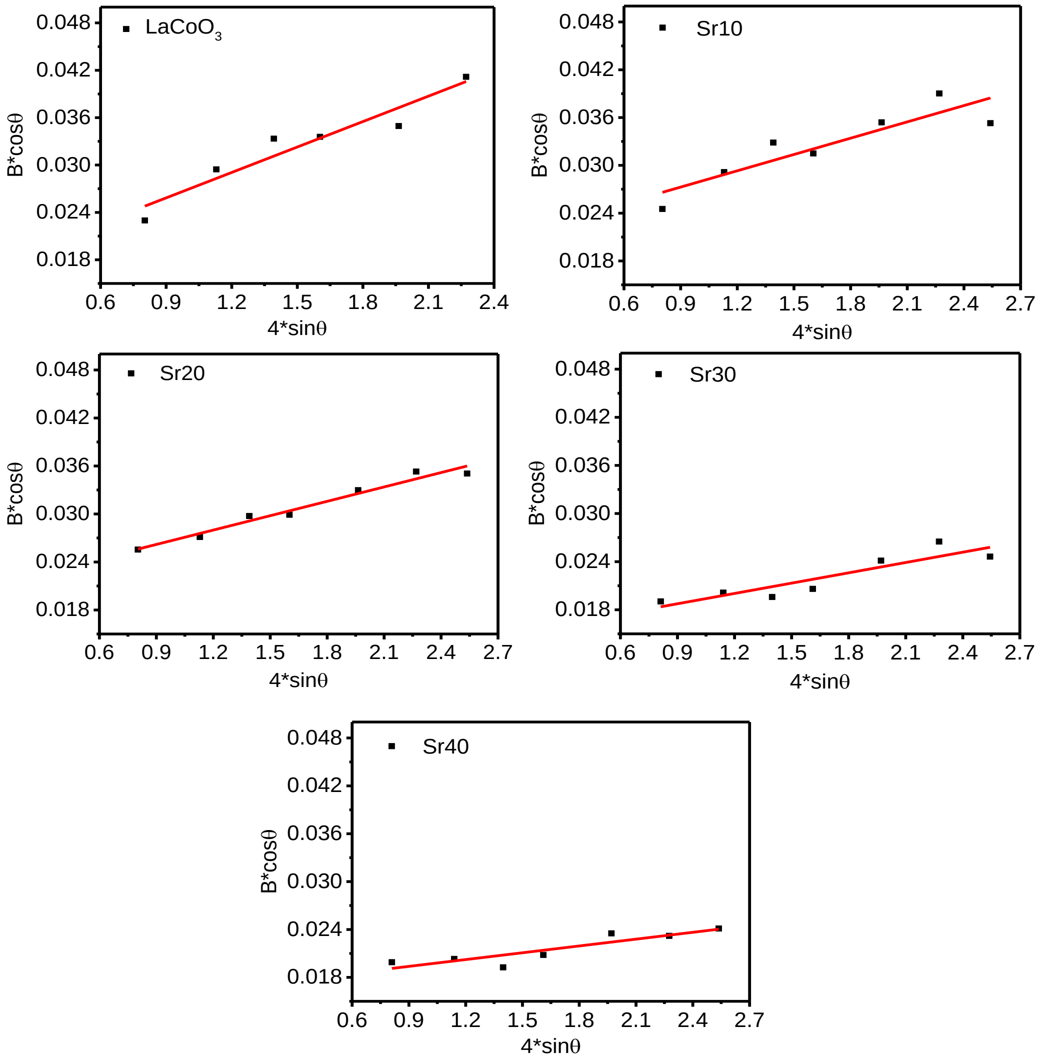


**Figure S1.** Williamson-Hall plots of $La_{1-x}Sr_xCoO_3$ samples. B indicates the integral breadth. The measured lattice strain values for LCO, Sr10, Sr20, Sr30 and Sr40 are 0.0107, 0.0068, 0.0059, 0.0043 and 0.0028, respectively.

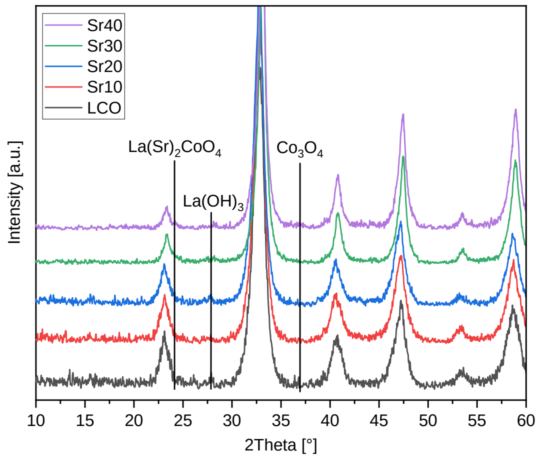


**Figure S2.** Stacked XRD patterns of $La_{1-x}Sr_xCoO_3$ samples, indicating the small diffraction peaks of the secondary phases. $La(OH)_3$ (ICSD: 31584), $Co_3O_4$ (ICSD: 36256) and $La_{2-x}Sr_xCoO_4$ (ICSD: 151635). Note, $La(OH)_3$ phases can likely contain a certain amount of Sr due to the substitution of La with Sr.

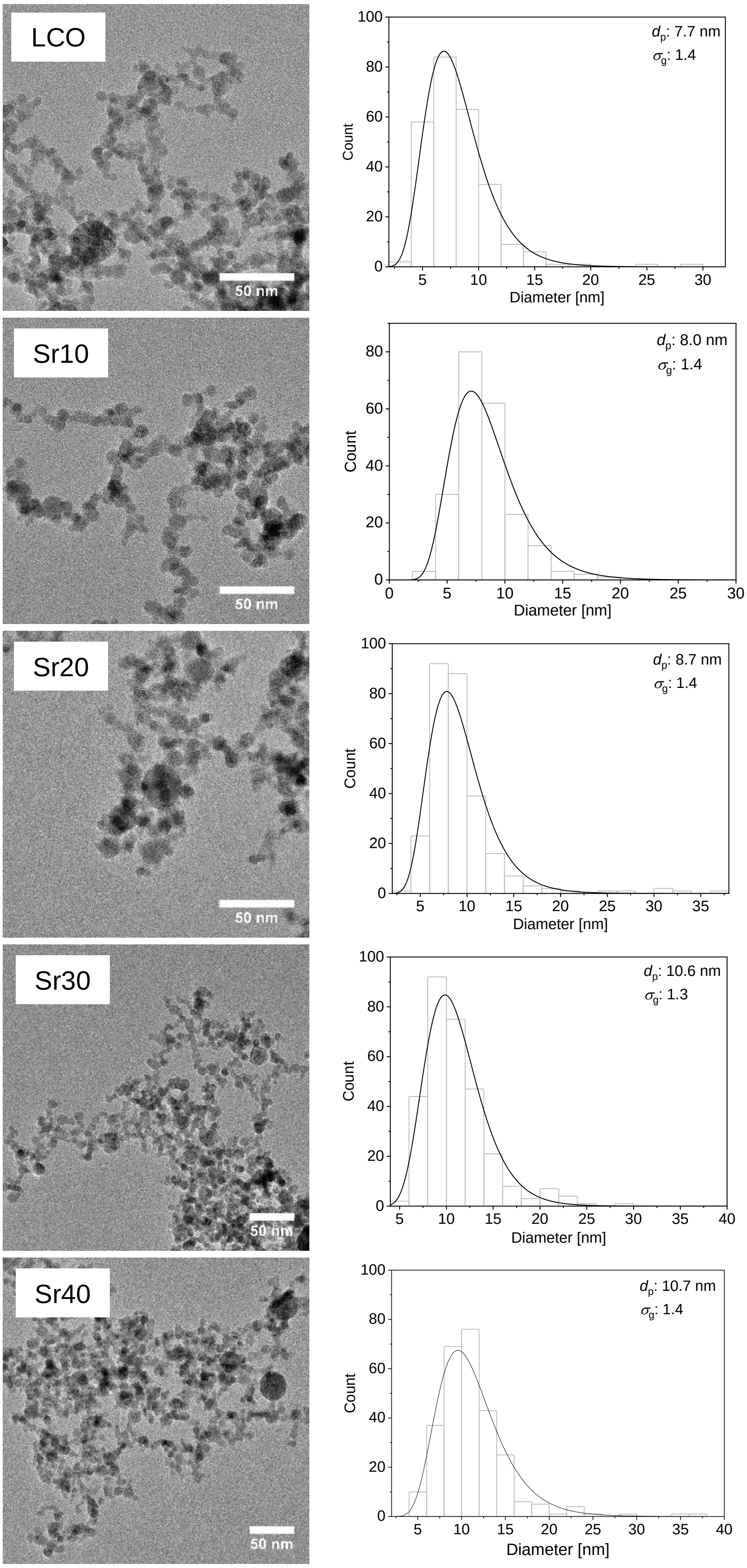


**Figure S3.** TEM images showing the particle size distributions and the histograms fitted with lognormal function in order to determine $d_p$ and $\sigma_g$.

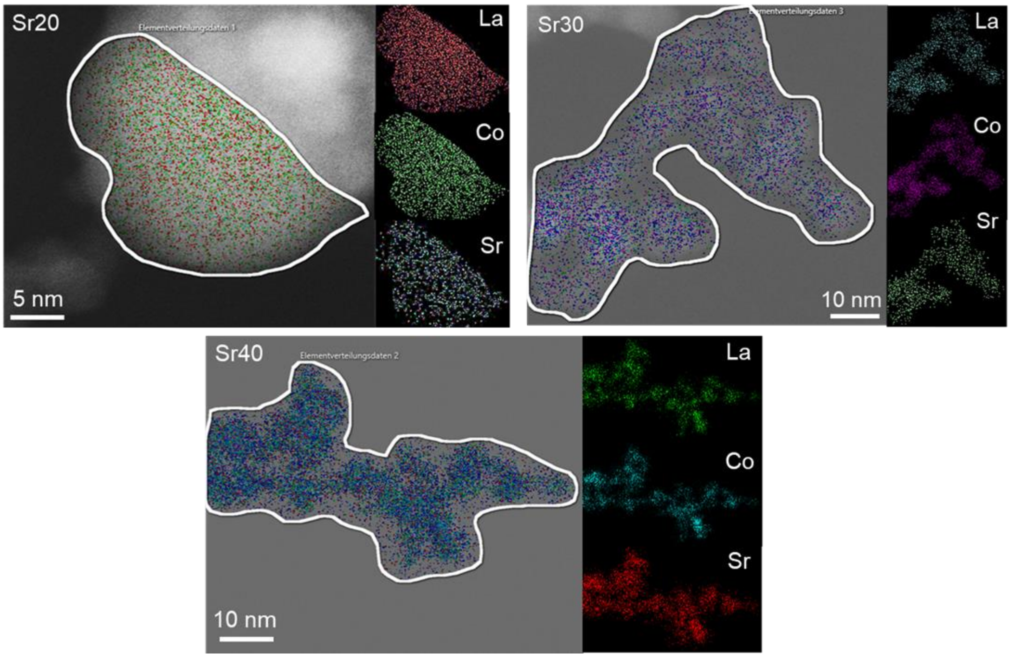


**Figure S4.** EDX elemental mapping of La, Co, and Sr in the Sr20, Sr30 and Sr40 samples at HAADF-STEM mode.

**Table S1.** The bulk composition of $La_{1-x}Sr_xCoO_3$ catalysts derived from the EDX measurements using the STEM mode.

| Catalysts | (La+Sr)/Co [-] | Sr/(Sr+La) [%] |
|---|---|---|
| LCO | 0.98 | - |
| Sr10 | 1.07 | 8.00 |
| Sr20 | 1.07 | 18.00 |
| Sr30 | 0.99 | 29.00 |
| Sr40 | 0.97 | 40.00 |

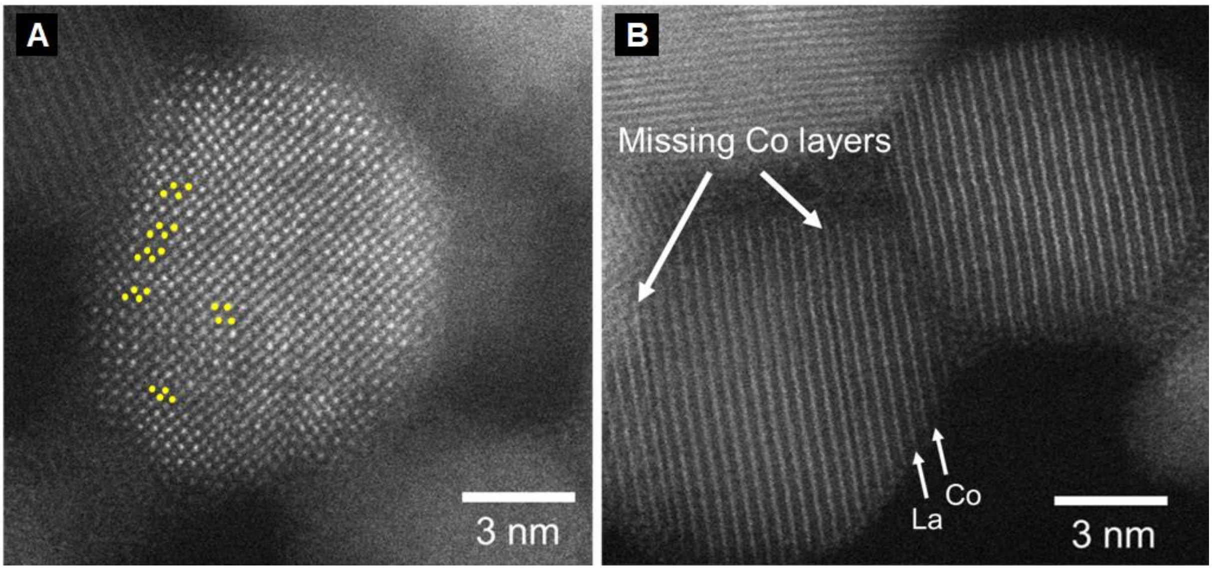


**Figure S5**. HRTEM images of LCO samples indicating the local Ruddlesden Popper-type antiphase boundaries (yellow circles, A) and Co-deficient layers (B) in the selected crystallites.

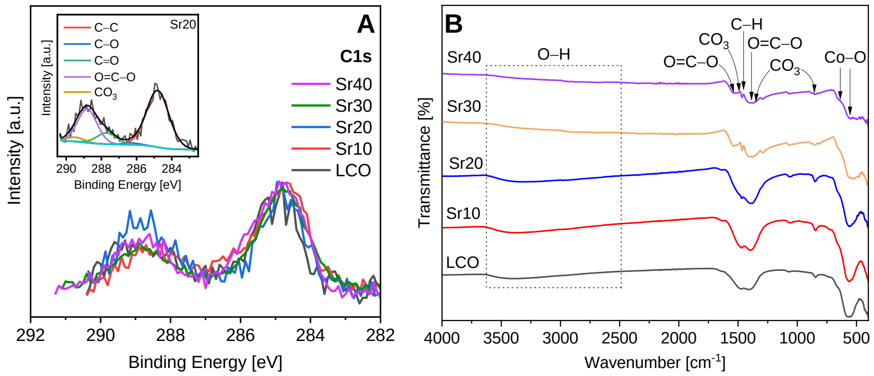


**Figure S6.** C 1s XP spectra (A) and ATR-FTIR spectra (B) of $La_{1-x}Sr_xCoO_3$ nanoparticles. Deconvoluted peaks are indicated in the inset figure of the C 1s figure.

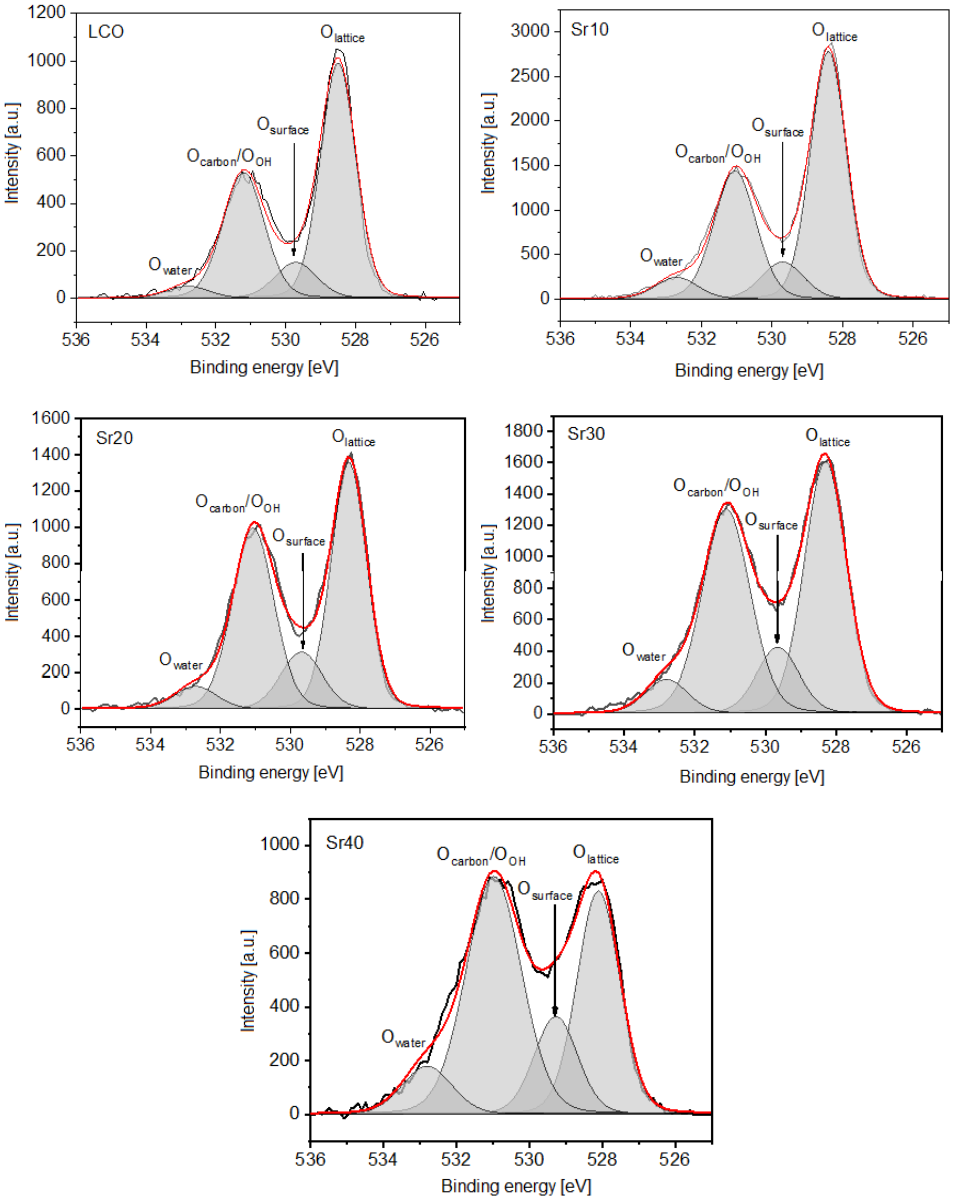


**Figure S7**. O 1s XP spectra of $La_{1-x}Sr_xCoO_3$ nanoparticles. $O_{surface}$ denotes $O_2^{2-}/O^-$ species. The deconvoluted peaks were obtained according to a previous study.[76]

The surface chemical groups in the C 1s and O 1s spectra were investigated (Figure S6 and S7). The oxygen-bound carbon groups appear in the C 1s spectra at 290-286 eV, being related to carbonaceous combustion residuals remaining in the nanoparticles. Slightly higher peaks of carbonate and carboxylate groups are visible in the XP spectrum of Sr20 compared with the other samples (Figure S6a). On the other hand, regarding the overall content of carbonaceous residuals, ATR-FTIR spectra in Figure S6b show that the intensities of C-O, C=O, O=C-O, $CO_3$ groups relative to those of Co–O are higher in Sr20, Sr30 and Sr40, indicating higher amounts of bulk carbon species for high Sr concentrations. Additionally, Figure S7 shows the deconvoluted O 1s XP peaks with the lattice oxygen species of the perovskite at 528.6 eV as well as $O_{surface}$ ($O_2^{2-}/O^-$) at 529.5 eV, $O_{carbon/OH}$ at 531.5 eV and $O_{water}$ at 533.0 eV. $O_{carbon/OH}$ intensities show an increase for Sr20, Sr30 and Sr40 in agreement with the band intensity of carbon-oxygen groups in the ATR-FTIR spectra. These peaks mainly correspond to $O_{carbon}$ species, as OH groups were not detected in the ATR-FTIR spectra. In addition, the $O_2^{2-}/O^-$ peak intensity increases for Sr30 and Sr40, which can be related to oxygen vacancies in the perovskite materials[61] as well as oxygen species of $Co_3O_4$.[62]

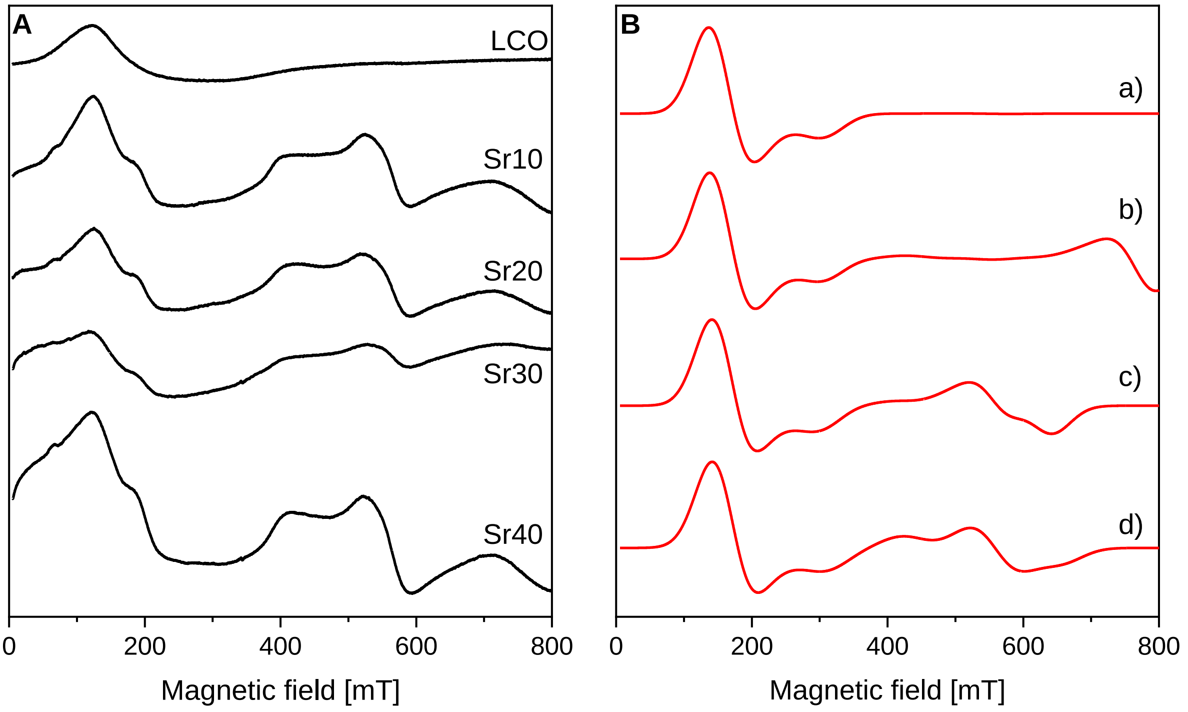


**Figure S8.** Experimental X-band cw EPR spectra of the $La_{1-x}Sr_xCoO_3$ samples recorded at 10 K (A) and simulations for high spin $Co^{2+}$ ions (S = 3/2) (B). These simulations were obtained with a spin Hamiltonian containing Zeeman term, with $g$ = 2.24, Gaussian linewidth broadening of 70 mT and zero field splitting (zfs) terms, with a) $D$ > 1.2 $cm^{-1}$ and $E/D$ = 0, b) $D$ = 0.6 $cm^{-1}$ and $E/D$ = 0, c) $D$ = 0.38 $cm^{-1}$ and $E/D$ = 0 and d) $D$ = 0.38 cm-1 and $E/D$ = 0.03 (B). It should be noted that Sr doped samples showed more $Co^{2+}$ signal in contrast to the LCO and the $Co^{2+}$ content increased in this order: LCO < Sr30 ≈ Sr20 < Sr10 <Sr40.

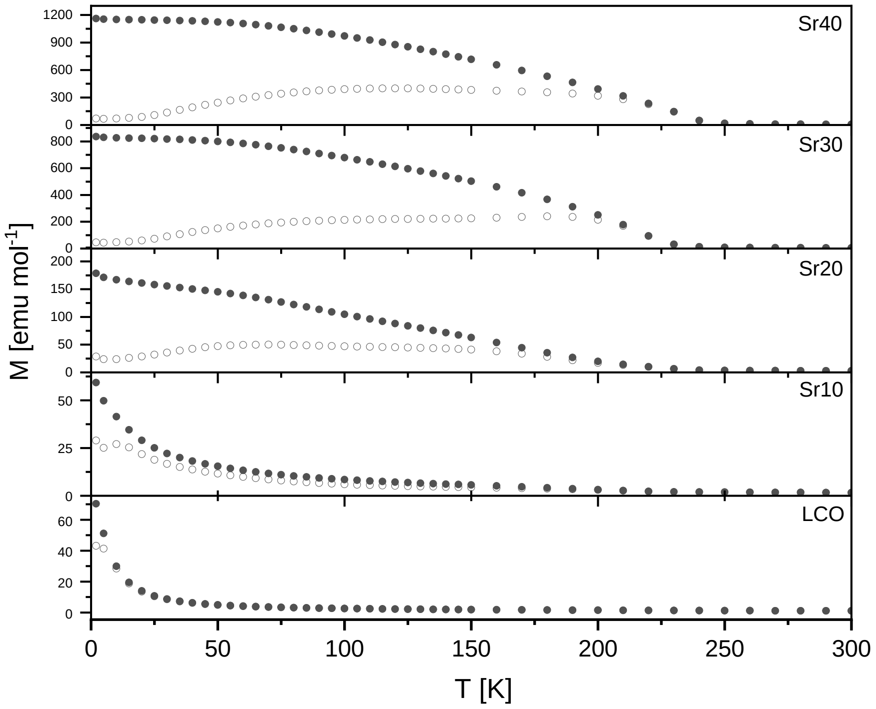


**Figure S9.** Field cooled (FC, 50 mT) and zero field cooled (ZFC) temperature dependent magnetization data for $La_{1-x}Sr_xCoO_3$ with Sr doping of $x$ = 0, 0.1, 0.2, 0.3, 0.4. From $\partial M/\partial T$, $T_C$ (Curie temperature, where the paramagnetic (PM) to ferromagnetic (FM) transition occurs) values of ~230 K ($x$ = 0.4), ~210 K ($x$ = 0.3) and ~170 K ($x$ = 0.2) were extracted. At lower Sr doping (x < 0.2) the FC magnetization shows a different temperature dependence, which was assigned to spin-glass (SG) behavior, with an onset of $x \leq 0.18$.[67]

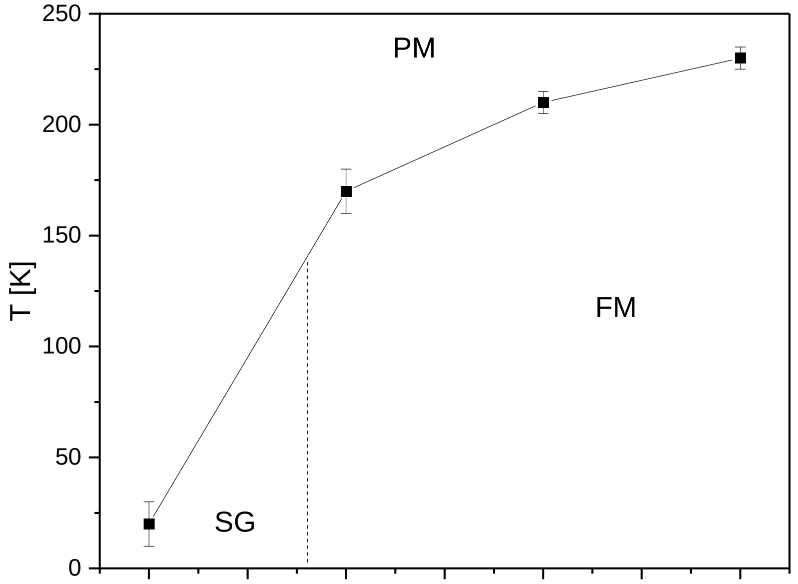


**Figure S10.** Magnetic phase $T$-$x$ diagram for $La_{1-x}Sr_xCoO_3$, with PM = paramagnetic phase, FM = ferromagnetic phase, SG = spin glass phase. The solid black squares indicate $T_C$ values derived from data plotted in Fig. S9. The dashed line indicates the critical doping level $x_c$ = 0.18, where the metal to insulator transition and the transition to a spin glass occurs.[67]

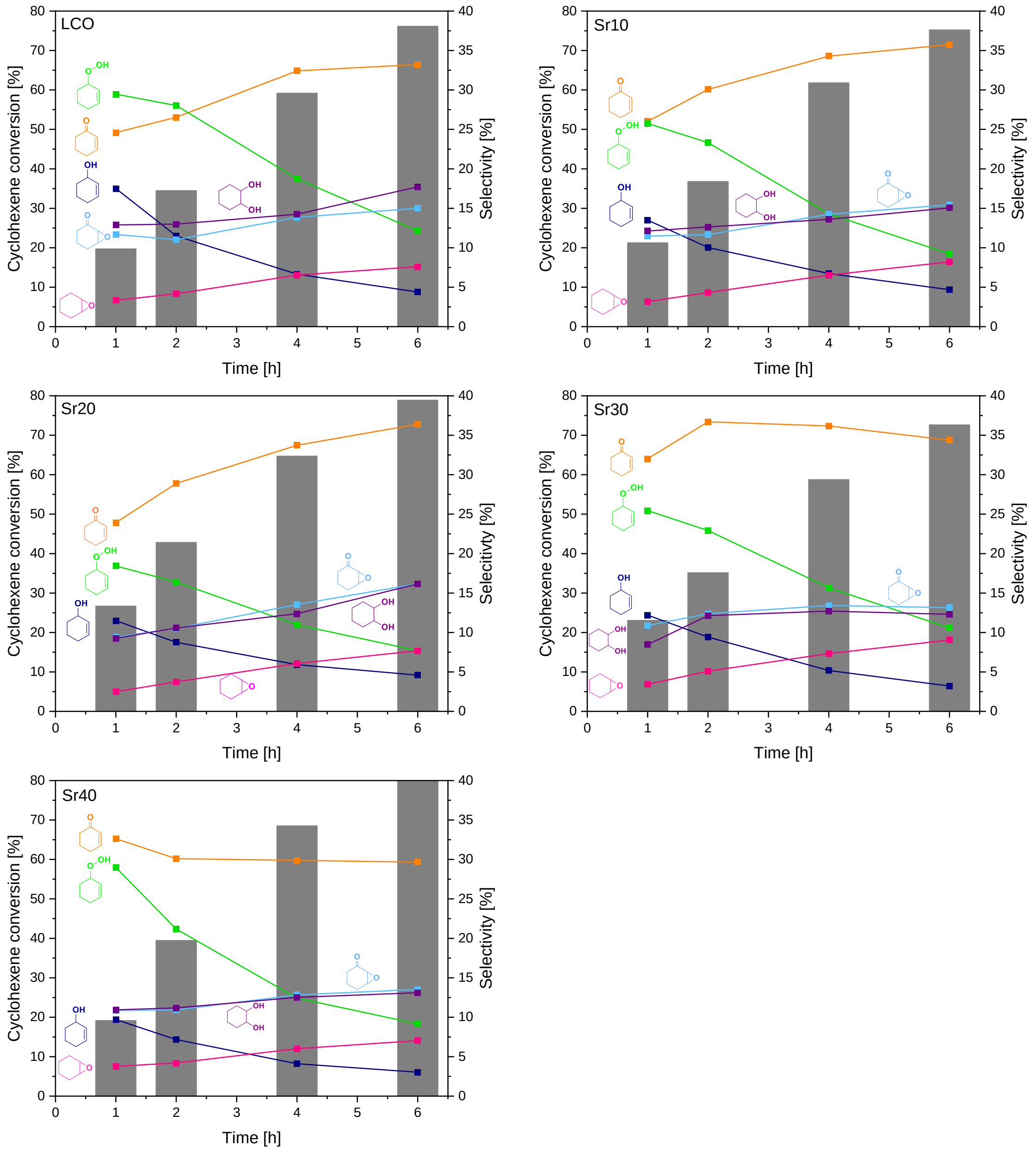


**Figure S11.** Effect of Sr amount on cyclohexene conversion and products selectivities over different $La_{1-x}Sr_xCoO_3$ catalysts under standard conditions.

**Table S2.** Comparison of product yields for cyclohexene oxidation over different $La_{1-x}Sr_xCoO_3$ perovskites after 6 h (allylic products: 2-cyclohexene-1-one, 2-cyclohexene-1-ol, 7-oxabicyclo[4.1.0]-heptane-2-one; epoxidation products: cyclohexane-1,2-diol, cyclohexene oxide; hydrogenation products: cyclohexane-1,2-dione).

| Sample | Yield [%] | | | |
|---|---|---|---|---|
| | Allylic products | Epoxidation products | Hydrogenation products | 2-Cyclohexene-1-hydroperoxide |
| LCO | 35.8 | 21.6 | 1.0 | 11.6 |
| Sr10 | 37.6 | 22.0 | 1.2 | 8.6 |
| Sr20 | 40.8 | 23.7 | 1.2 | 7.7 |
| Sr30 | 34.4 | 20.3 | 0.9 | 9.7 |
| Sr40 | 37.8 | 23.8 | 1.3 | 10.6 |

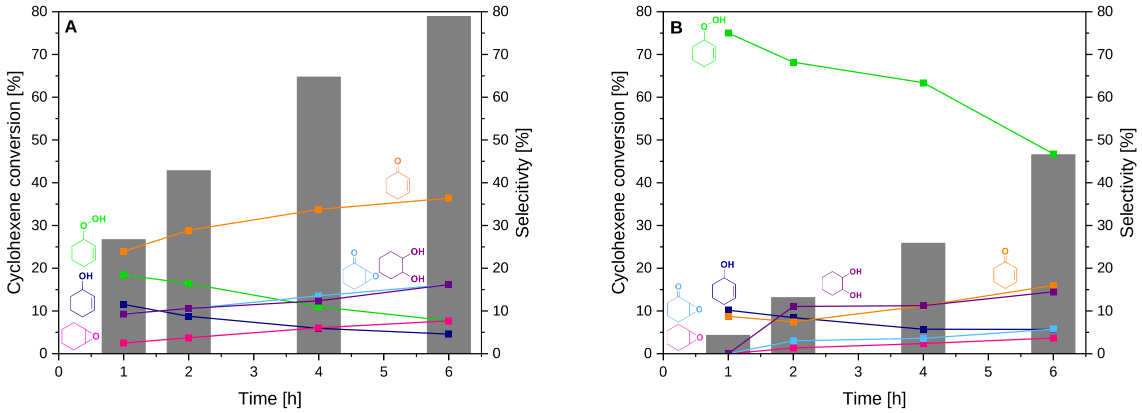


**Figure S12.** Cyclohexene oxidation under standard conditions over Sr20 (A) and over a commercial $La_{0.8}Sr_{0.2}CoO_3$ catalyst (B).

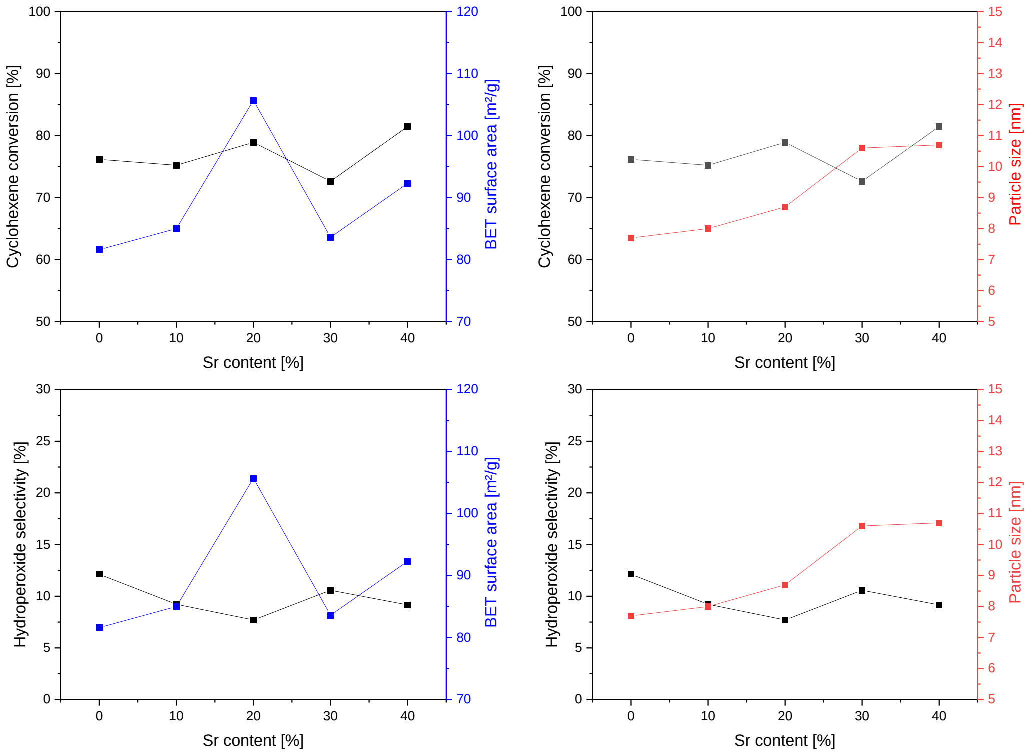


**Figure S13.** Correlations of cyclohexene conversion (above) and hydroperoxide selectivity (below) after 6 h with specific surface area (left) and particle size (right) of the $La_{1-x}Sr_xCoO_3$ perovskites.

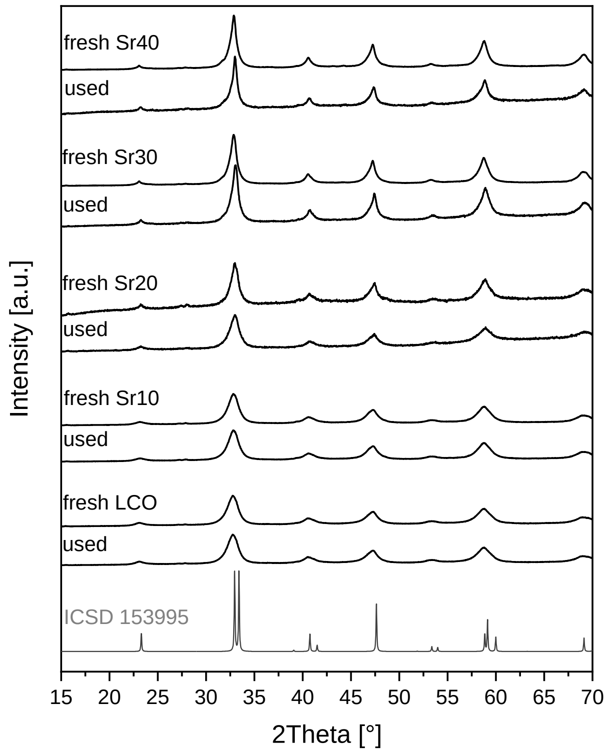


**Figure S14.** XRD patterns of fresh and used $La_{1-x}Sr_xCoO_3$ catalysts after cyclohexene oxidation under standard conditions.

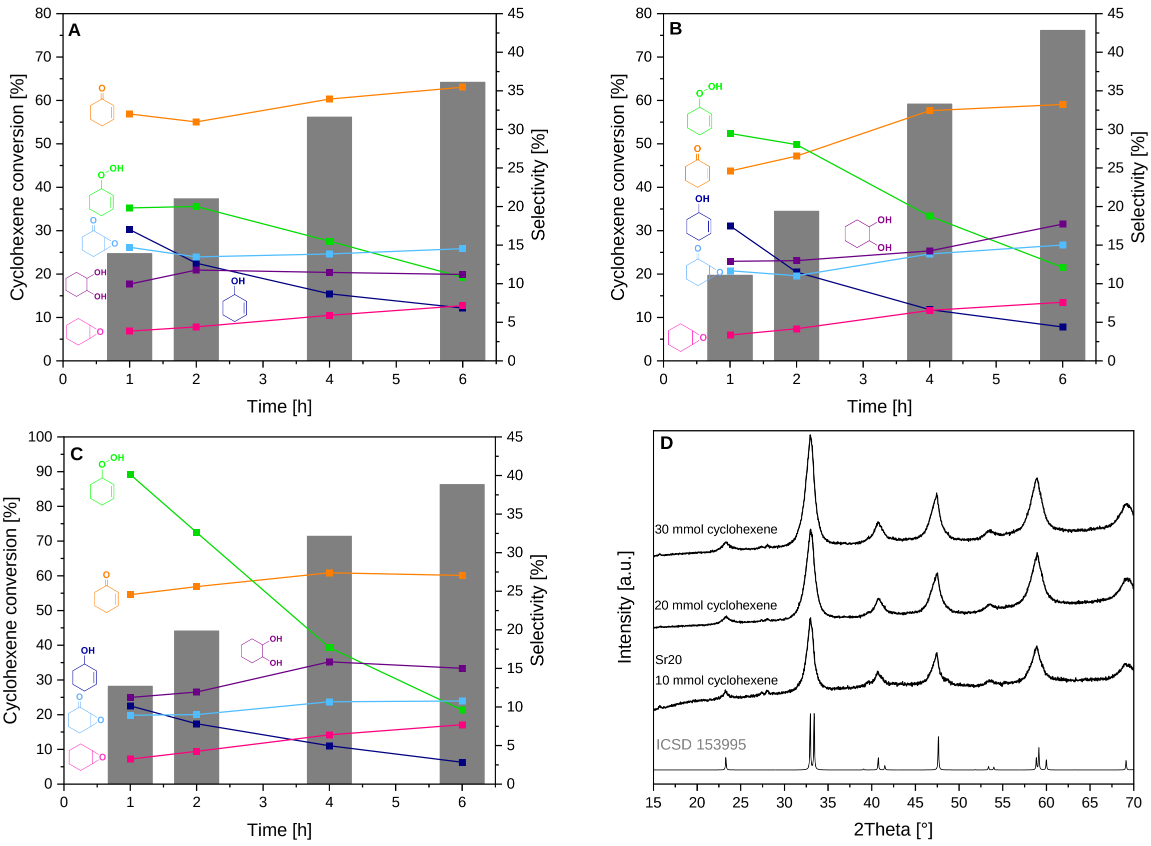


**Figure S15.** Effect of initial cyclohexene concentration of 10 mmol (A), 20 mmol (B) and 30 mmol (C) on cyclohexene oxidation over Sr20 and XRD patterns of spent catalysts (D).

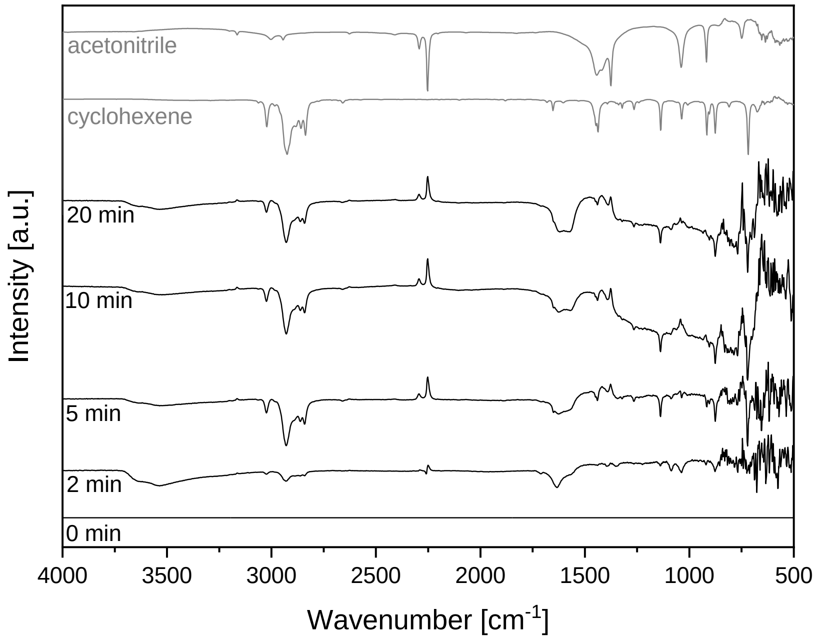


**Figure S16.** Time-dependent ATR-IR spectra of Sr20 in the presence of acetonitrile and cyclohexene at 60 °C. Reference spectra of acetonitrile and cyclohexene are shown in grey. The time-dependent spectra are difference spectra, of which the initial spectrum of pure acetonitrile was subtracted, showing the evolving cyclohexene bands and the vanishing acetonitrile bands.

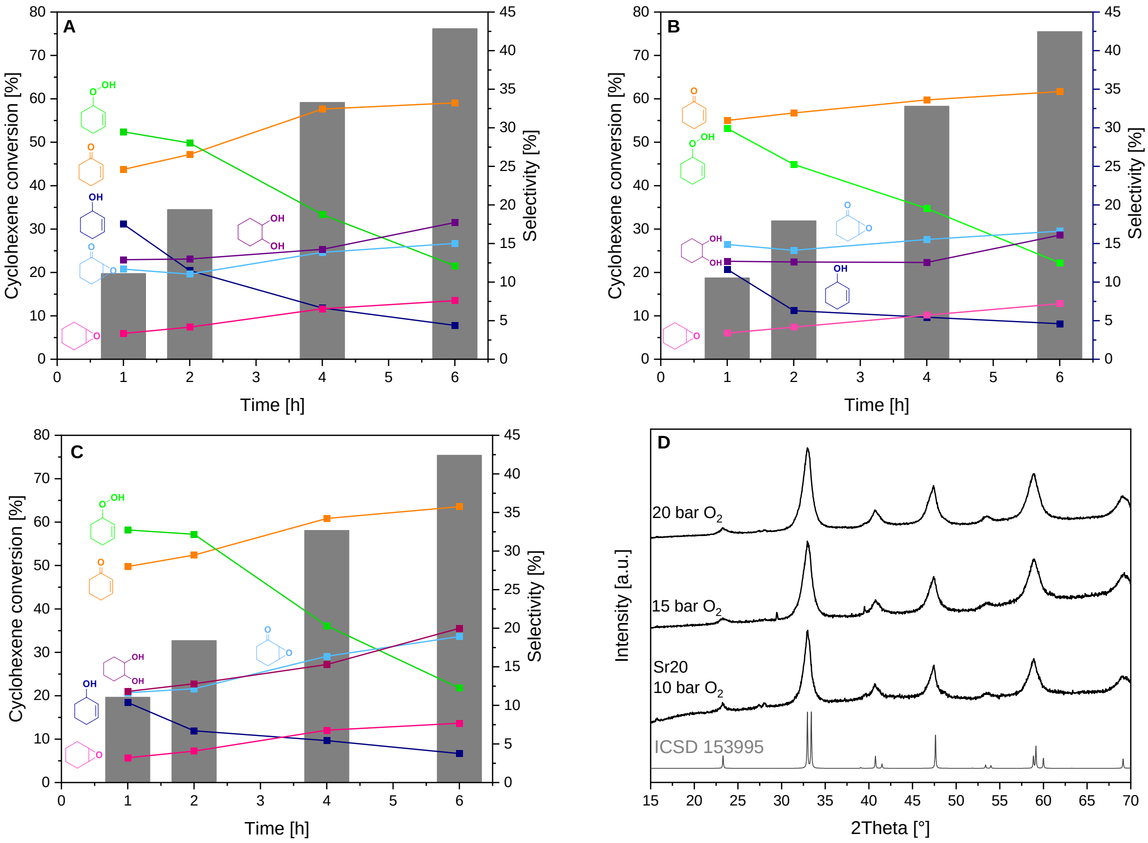


**Figure S17.** Effect of $O_2$ pressure on cyclohexene oxidation over Sr20 at 10 bar (A), 15 bar (B) and 20 bar (C) and XRD patterns of used catalysts (D).

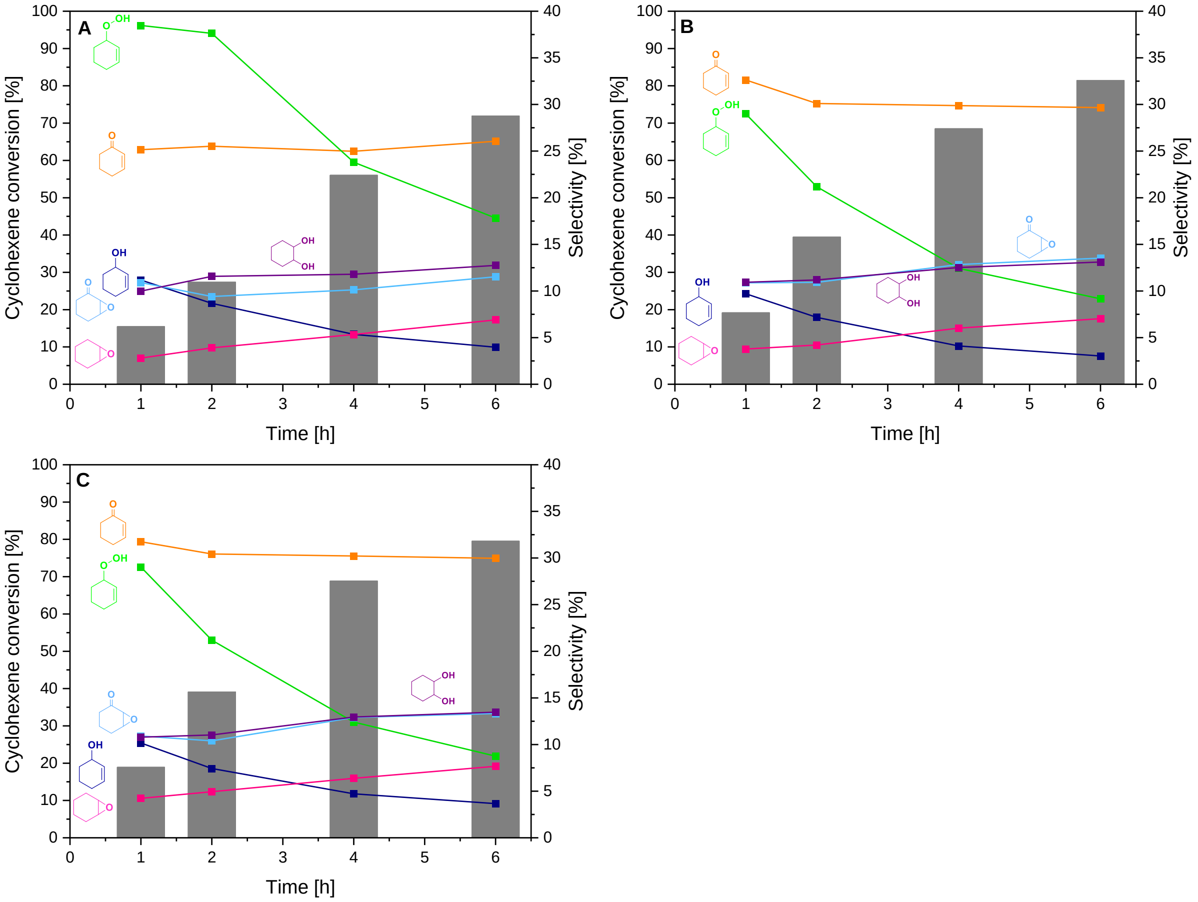


**Figure S18.** Effect of stirring speed on cyclohexene oxidation over Sr40 at 450 rpm (A), 600 rpm (B) and 750 rpm (C).

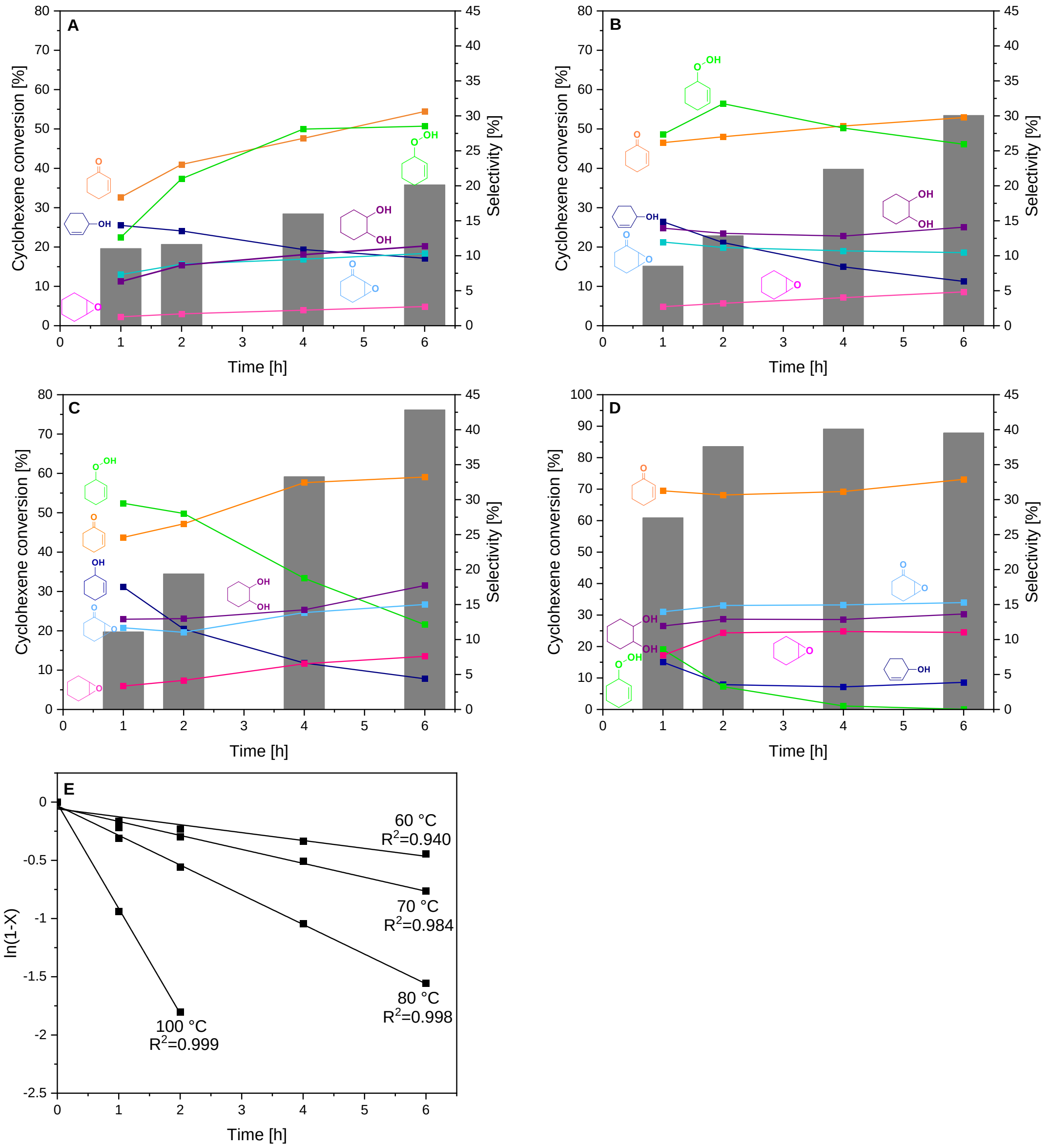


**Figure S19.** Effect of temperature on cyclohexene oxidation over Sr20 at 60 °C (A), 70 °C (B), 80 °C (C) and 100 °C (D) and the linearized plot of cyclohexene conversion as a function of time (E).

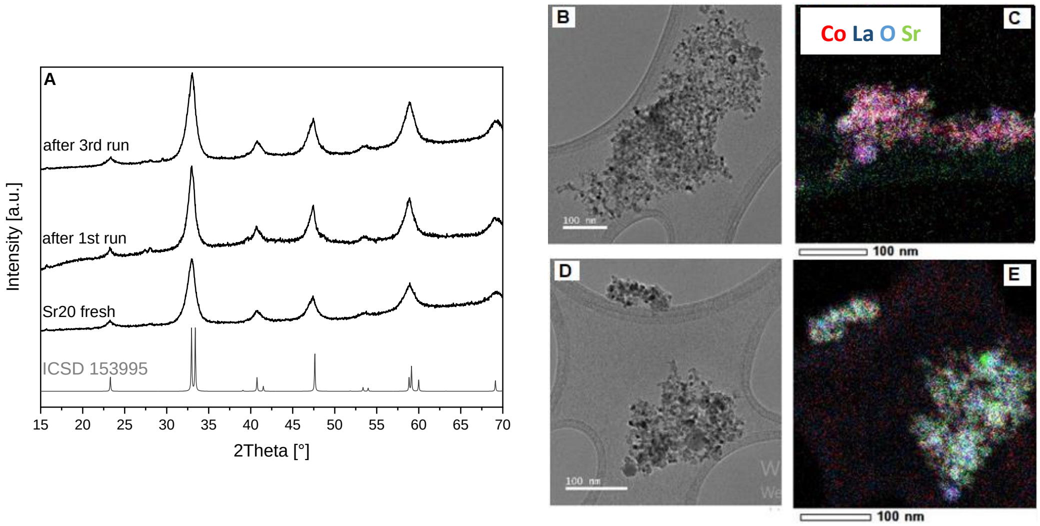


**Figure S20.** Reusability test for cyclohexene oxidation over Sr20. XRD patterns of fresh catalyst und spent one after the first and third run (A), STEM image (B) and EDX mapping (C) of fresh Sr20; STEM image (D) and EDX mapping (E) of Sr20 after three reaction runs.

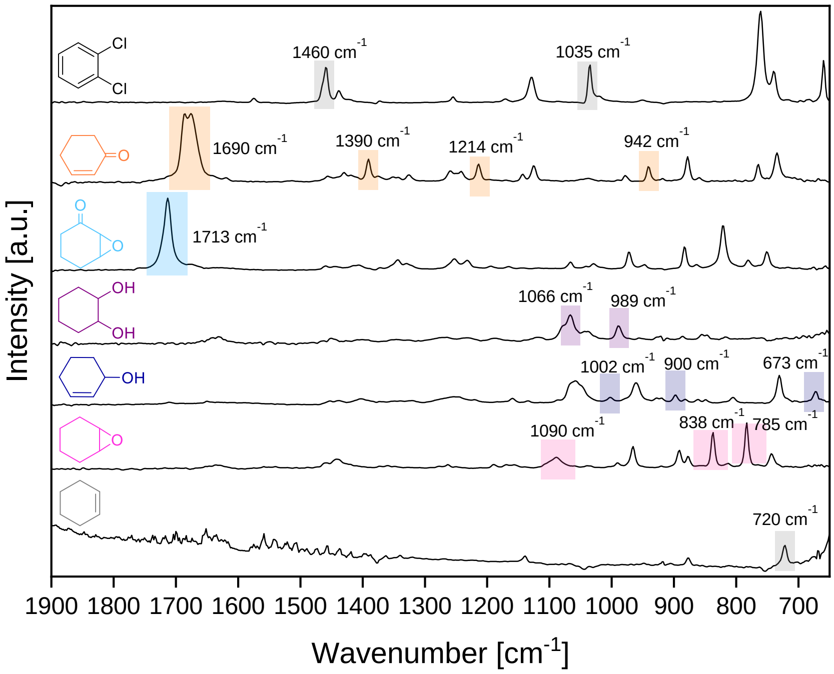


**Figure S21.** ATR-IR spectra of standard compounds in acetonitrile.

**Table S3.** The assigned vibrations for reference samples.

| Compound | Characteristic vibration [$cm^{-1}$] | Type of vibration[51] |
|---|---|---|
| 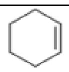 | 720 | cis alkene $sp^2$ C-H bend |
| 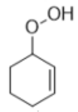 | 949 | R-O-O-H |
| 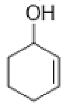 | 1002 | alkoxy C-O stretch |
| 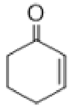 | 1690 | C=O stretch |
| 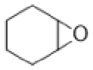 | 1090 | alkoxy C-O stretch |
| 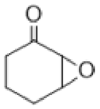 | 1713 | C=O stretch |
| 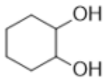 | 1066 | alkoxy C-O stretch |

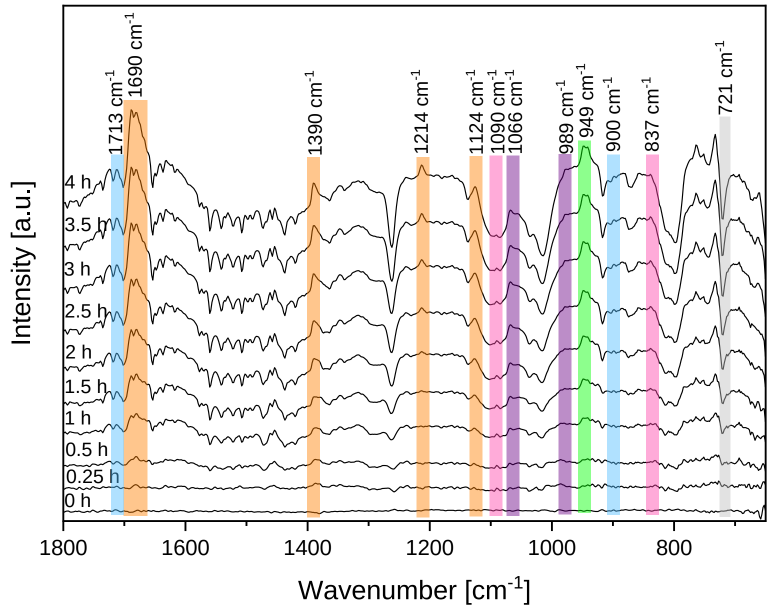


**Figure S22.** Untreated *in situ* ATR-IR spectra for the oxidation of cyclohexene over Sr40 catalyst in the range of 1800 to 650 $cm^{-1}$ after background subtraction. The spectrum for background subtraction is taken of the mixture of acetonitrile, *o*-dichlorobenzene as internal standard for GC analysis, cyclohexene and Sr40 catalyst under $N_2$ atmosphere at 80 °C.

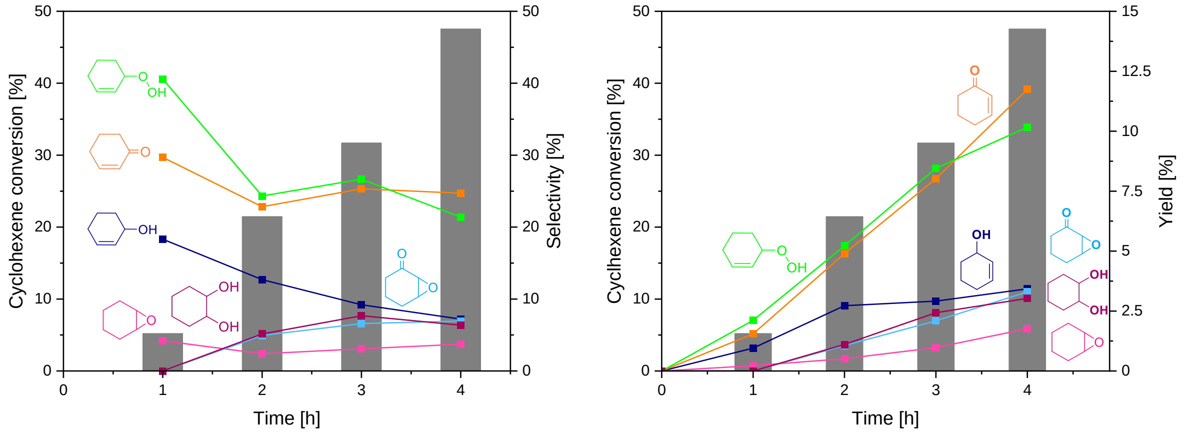


**Figure S23.** Quantitative offline GC analysis of cyclohexene oxidation over Sr40 during *in situ* ATR-IR spectroscopy.

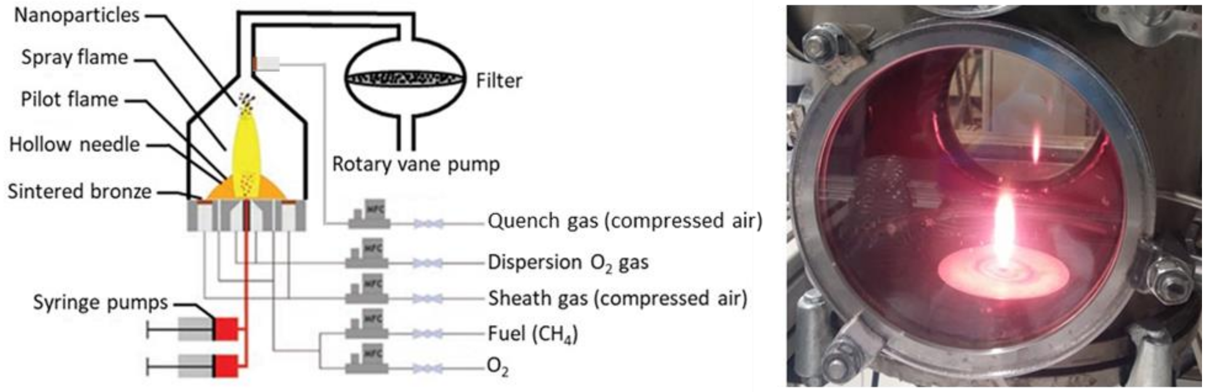


**Figure S24.** Schematic of the spray-flame reactor (left) and a picture of the spray flame (right), observed during the synthesis of the $La_{1-x}Sr_xCoO_3$ mixed oxides.

**Table S4.** Operating conditions of the spray-flame process and precursor solutions used for the synthesis of mixed $La_{1-x}Sr_xCoO_3$ perovskites.

| $La_{1-x}Sr_xCoO_3$ | x= 0, 0.1, 0.2 | x= 0.3, 0.4 |
|---|---|---|
| $CH_4/O_2$ pilot flame [slm/slm] | 2/4 | 2/4 |
| Dispersion gas $O_2$ [slm] | 6 | 7 |
| Solution flow rate [ml/min] | 3 | 4 |
| Pressure [mbar] | 930 | 930 |
| Sheath gas (compressed air) [slm] | 170 | 170 |
| Quench gas (compressed air) [slm] | 170 | 170 |
| Precursors | La-, Co-acetate hydrates, Sr-acetate | La-, Co-acetate hydrate, Sr-acetate |
| Solvent Mixtures [vol. %] | Propionic acid:1-propanol (2:3) | Propionic acid:1-propanol (2:3) |
| (La+Sr) and Co concentration [M] | 0.2 | 0.2 |

## Experimental Section

*Materials*

$La_{1-x}Sr_xCoO_3$ nanoparticles were synthesized using the metal precursors of lanthanum(III) acetate hydrate ($La(CH_3COO)_3$ • x $H_2O$, 99.9 %), cobalt(II) acetate tetrahydrate ($Co(CH_3COO)_2$ • 4 $H_2O$, >98 %) and strontium(II) acetate ($Sr(CO_2CH_3)_2$, > 99%) from Sigma-Aldrich. As solvents, propionic acid (>99 %) and 1 propanol (anhydrous, 99.7 %) were used.

A commercial $La_{0.8}Sr_{0.2}CoO_3$ catalyst was supplied by Cerpotech.

For cyclohexene oxidation, cyclohexene (99 %), 2-cyclohexene-1-one (98 %), 2-cyclohexene-1-ol (95 %), cyclohexene oxide (98 %), 7-oxabicyclo[4.1.0]heptan-2-one (98 %), cyclohexan-1,2-diol (98 %) and 1,2-dichlorobenzene (99 %) were purchased from Sigma Aldrich. Acetonitrile in analytical reagent grade was bought from Fisher Chemicals. All reagents were employed without further purification.

*Catalyst Synthesis*

Spray-flame synthesis of perovskite nanoparticles was performed in a homemade reactor which allows full control of gas flow and pressure. The reactor setup was described previously,[24c,26a,52] and its scheme is shown in Figure S24. The process parameters and the precursor/solvent compositions used for the syntheses are tabulated in Table S4. In brief, propionic acid was mixed with 1-propanol in a glass flask at room temperature and stirred for 5 min. The La-, Co-, and Sr-precursors were subsequently added to the solvent mixture and the flask was heated in a silicon oil bath at 60 °C to dissolve the precursors under constant magnetic stirring for at least 1 h. The total metal concentration of (La+Sr) and Co was adjusted to 0.2 M. The nominal atomic ratios of Sr/(La+Sr) (xSr) were adjusted to 0, 0.1, 0.2, 0.3, and 0.4.

Afterwards, the precursor solution was transferred into the reactor where the dispersion $O_2$ gas atomizes the precursor solution. For $LaCoO_3$, $La_{0.9}Sr_{0.1}CoO_3$ and $La_{0.8}Sr_{0.2}CoO_3$, the dispersion $O_2$ gas of 6 slm with a precursor flow rate of 3 mL/min was used. For $La_{0.7}Sr_{0.3}CoO_3$ and $La_{0.6}Sr_{0.4}CoO_3$, the gas to liquid volume ratios was reduced by the dispersion $O_2$ gas of 7 slm with a precursor flow rate of 4 mL/min being used. Premixed pilot flame ($CH_4/O_2$) surrounding the hollow needle was used to ignite and stabilize the spray flame while a sheath gas surrounding the flame was used to stabilize the reactor gas flow (Figure S24). The reactor was operated at a pressure of 925-930 mbar. The particles formed were collected on a circular filter membrane from the burner off-gases.

*X Ray Diffraction (XRD) Measurements*

X-ray diffraction (XRD) patterns were measured with a PANalytical X'Pert PRO X-ray diffractometer operated at 40 kV and 40 mA using Ni-filtered Cu-Kα radiation and a linear position sensitive X'Celerator detector in combination with a monochromator. A silicon single crystal was used as sample holder and the diffraction data of as-prepared nanoparticles were measured in the 2θ range from 10–65° with a step size of 0.03° and a scan speed of 0.05°/min. The average crystal size and microstrain of the nanoparticles was estimated by the Williamson-Hall method. As octahedral-shaped crystals were mostly observed by TEM measurements, the Scherrer constant of octahedral crystallites (i.e., Kβ ~ 1.1[53]) was used as shape factor.

*$N_2$ Physisorption*

$N_2$ physisorption measurements were performed at 77 K in a Quantachrome instruments apparatus. The as-prepared powders were firstly degassed at 100 °C under vacuum for 5 h to remove adsorbed water. The specific surface areas were determined from the adsorption isotherm using the BET method. The bulk density values were taken from ICSD data files of $La_{1-x}Sr_xCoO_3$.

*High Resolution Transmission Electron Microscopy (HR TEM)*

High-resolution and high angle annular dark-field scanning transmission electron microscopy (HR-TEM, HAADF-STEM), and energy dispersive X ray spectroscopy (EDX) were performed at a JEM-2200FS (JEOL, Akishima, Japan). For sample preparation, nanoparticles were dispersed in ethanol for 5 min with ultrasonic treatment, and the dispersions of the nanoparticles were dropped on a carbon-coated copper grid for TEM measurements. For

estimating count mean particle diameters (CMDs), the diagonal distance of 400 different particles per sample were measured. The size histograms were fitted with lognormal curves to determine CMDs.

*X Ray Photoelectron Spectroscopy (XPS)*

VersaProbe II (Ulvac-Phi, Chanhassen, USA) was used to measure XP spectra of the nanoparticles. Al $K_\alpha$ radiation ($hv$= 1253.6 eV) with a pass energy of 11.75 eV was used to obtain the Co 2p, La 3d, Sr 3d, C 1s, and O 1s XP spectra. The binding energies of all spectra were corrected to C 1s at 284.8 eV. For the deconvolution of Co 2p and Sr 3d spectra, the binding energies and FWHM reported in the literature were taken into account.[32a,54] Deconvolution of O 1s spectra was carried out referring to a previous report.[36] The deconvolution of C 1s spectra and the calculation of carbon-bound oxygen intensity in the O 1s spectra were realized using the method described in the literature.[55]

*Superconducting Quantum Interference Device (SQUID) Magnetometry*

The temperature-dependent magnetization of $La_{1-x}Sr_xCoO_3$ was measured from powder samples of solid material immobilized in eicosane by using a superconducting quantum interference device (SQUID, MPMS-7, Quantum Design, calibrated with a standard palladium reference sample, error < 2 %) with a field of 50 mT (field cooled samples) or 0 T (zero field cooled) in the temperature range from 2 to 300 K.

*Gas-Phase Oxidation of 2-Propanol*

2-Propanol oxidation was carried out in a stainless steel microreactor setup. 10 mg catalyst powders of the sieve fraction 250-355 µm were filled in a glass lined stainless steel U-tube reactor. To minimize the effect of hot spots, the samples were diluted with 50 mg quartz pearls of the same size. A thermocouple was directly placed into the catalyst bed to control the temperature. Passing He through a saturator at 273 K enabled 2-propanol dosing. By using mixing valves, 2-propanol/He and $O_2$ were mixed and diluted with He. For 2-propanol oxidation, 0.18 % 2-propanol/ 0.18 % $O_2$/ He feed gas was passed over the catalyst for 1 h and the sample was heated to 573 K with a heating rate of 0.5 K $min^{-1}$. A calibrated quadrupole mass spectrometer (QMS, Balzers GAM422) was used for time-resolved quantitative online gas analysis.

*Catalytic Oxidation Reactions*

Oxidation reactions were carried out in a 100 mL autoclave reactor equipped with a Teflon liner (Parr Instruments). 60 mg catalysts were dispersed in 30 mL acetonitrile. 20 mmol cyclohexene and 4.5 mmol 1,2-dichlorobenzene as the internal standard for GC analysis were added. The autoclave was purged with $O_2$ for three times and pressurized to 10 bar. Subsequently, the reaction mixture was heated to 80 °C. The reaction was initiated by switching on the stirrer to 600 rpm at 75 °C. Equally, stirring was started 5 °C below the set temperature for oxidation reactions performed at different temperatures. Samples were taken through an online sampling system after 1, 2, 4, and 6 h. For GC analysis, two samples of 1.5 mL were taken after filtering off the catalyst by a syringe filter (200 nm). One sample was purely analyzed while the other was treated with 2 mmol triphenylphosphine ($PPh_3$) to decompose the formed 2-cyclohexene-1-hydroperoxide into the corresponding alcohol 2-cyclohexene-1-ol. The hydroperoxide amount was calculated by subtraction of the detected alcohol amounts. The errors of the individual measurements were calculated by using the Gauss law for propagation of error considering the standard deviations of the GC measurements and of the calibration.

*Catalyst Reusability*

To test the reusability of the catalyst, three reaction runs were carried out under standard conditions. After each run, the catalyst was separated by centrifugation, washed three times with 5 mL acetonitrile, and dried overnight at room temperature. The catalyst amount decreased from initially 60 mg, to 56 and 51 mg in the second and third run, respectively.

*EPR Spectroscopy*

X-band cw EPR spectra were obtained using an X-band Bruker Elexsys E500 EPR spectrometer equipped with a ER4116DM dual mode resonator and an ESR 900 He cryostat. $La_{1-x}Sr_xCoO_3$ were measured as powder samples

(~3 mg) for detection of $Co^{2+}$. The EPR spectra were obtained at 10 K using a microwave frequency of ~9.64 GHz, microwave power of 63.15 mW, 800 mT field sweep centred at 405 mT, a Lock-In modulation amplitude of 0.7 mT, a time constant of 40.96 ms and a sweep time of 167.77 s, 4096 points and a modulation frequency of 100 kHz. The Co(II) simulations were obtained using EasySpin 5.2.30[56] with "pepper" for powdered samples in frozen state.

For the EPR spin trapping experiments, cyclohexene oxidation was carried out for 3 h under otherwise standard conditions. After 3 h of reaction, a sample was taken and the catalyst was immediately filtered off. Afterwards, the sample was directly transferred into an EPR tube containing 10 mg DMPO and frozen in liquid nitrogen.

The EPR spectra were obtained at 295 K for spin trapping experiments using a microwave frequency of ~9.64 GHz, microwave power of 20 mW, 10 mT field sweep centred at 337 mT, a Lock-In modulation amplitude of 0.1 mT, a time constant and a sweep time of 40.96 ms, 1024 points and a modulation frequency of 100 KHz. All simulations were obtained using EasySpin 5.2.30[56] with the function "garlic" for isotropic spectra at 295 K.

*ATR-IR Spectroscopy*

ATR-IR spectra were recorded using a Thermo Scientific 6700 FTIR spectrometer. A home-made ATR cell described elsewhere[57] was used with a Ge IRE (Korth Kristalle GmbH). 10 mg Sr20 was suspended in 1 mL distilled water and sonicated for 15 min. The suspended sample was deposited on the IRE by drop coating and the solvent was evaporated by heating to 80 °C. The deposit-evaporate procedure was repeated. Spectra were recorded with 256 scans in the range from 4000 – 500 $cm^{-1}$ and a resolution of 4 $cm^{-1}$. The ATR cell was heated to 60 °C during the measurements. The catalyst film in contact with 1 atm air was used as background. For the measurement, acetonitrile was filled into the ATR cell and spectra were recorded. Afterwards, cyclohexene (0.67 mol $L^{-1}$) was added and further spectra were recorded. Reference spectra of acetonitrile and cyclohexene were obtained according to the experimental procedure by using an uncoated Ge IRE.

*In situ* ATR-IR spectroscopy was carried out with a ReactIR 15 from Mettler Toledo equipped with a diamond internal reflection element (IRE), an AgX 6 mm x 1.5 m fiber probe, and a liquid nitrogen-cooled mercury-cadmium-tellurid (MCT) detector. The spectral range of 3000 to 650 $cm^{-1}$ was recorded with a resolution of 4 $cm^{-1}$.

For the *in situ* ATR-IR study, 200 mg Sr40 were suspended in 150 mL acetonitrile. 100 mmol cyclohexene and 22.5 mmol 1,2-dichlorobenzene as internal standard for GC analysis were added. The reaction solution was filled in a Teflon liner of a 350 mL autoclave (Berghof) equipped with an ATR-IR probe (Mettler Toledo). The reactor was flushed three times with nitrogen and pressurized to 3 bar. It was heated in the inert atmosphere to 80 °C under stirring at 300 rpm. After the temperature was stabilized, the oxidation reaction was initiated by pressurizing the reactor with oxygen to 20 bar. The reaction progress was monitored by recording the spectra every 2 min. Meanwhile, samples were taken through an online sampling system after 0, 1, 2, 3, and 4 h and anayzed by GC.

*Gas chromatographie*

Gas chromatography analysis was carried out in a 7820 A GC from Agilent Technologies. It was equipped with an Agilent DB-XLB column (30 m x 0.18 mm x 0.18 µm) and an FID detector. The injection volume was set to 0.5 µL with a split ratio of 75:1, a split flow of 30 mL/min and an inlet temperature of 260 °C. The column was first kept at 80 °C for 5 min. Subsequently, the oven was heated to 170 °C with a rate of 15 °C/min. Afterwards, it was heated with a ramp of 30 °C/min up to 300 °C to avoid deposits of the $PPh_3$ in the column. The end temperature was kept for 1 min.
